# Dysregulated gene subnetworks in breast invasive carcinoma reveal novel tumor suppressor genes

**DOI:** 10.1038/s41598-024-59953-0

**Published:** 2024-07-08

**Authors:** Shivangi Agarwal, Monalisa Parija, Sanoj Naik, Pratima Kumari, Sandip K. Mishra, Amit K. Adhya, Sushil K. Kashaw, Anshuman Dixit

**Affiliations:** 1 Department of Pharmaceutical Sciences, Dr. Harisingh Gour University, Sagar, 470003 India; 2 Institute of Life Sciences, Nalco Square, Bhubanesawar, 751023 Odisha India; 3 All India Institute of Medical Sciences, Bhubanesawar, 751019 India

**Keywords:** TNBC, ER/PR+/HER-2−, ER/PR−/HER-2+, Hotnet2 clusters, Western Blot, Immunohistochemistry, Computational biology and bioinformatics, Oncology

## Abstract

Breast invasive carcinoma (BRCA) is the most malignant and leading cause of death in women. Global efforts are ongoing for improvement in early detection, prevention, and treatment. In this milieu, a comprehensive analysis of RNA-sequencing data of 1097 BRCA samples and 114 normal adjacent tissues is done to identify dysregulated genes in major molecular classes of BRCA in various clinical stages. Significantly enriched pathways in distinct molecular classes of BRCA have been identified. Pathways such as interferon signaling, tryptophan degradation, granulocyte adhesion & diapedesis, and catecholamine biosynthesis were found to be significantly enriched in Estrogen/Progesterone Receptor positive/Human Epidermal Growth Factor Receptor 2 negative, pathways such as RAR activation, adipogenesis, the role of JAK1/2 in interferon signaling, TGF-β and STAT3 signaling intricated in Estrogen/Progesterone Receptor negative/Human Epidermal Growth Factor Receptor 2 positive and pathways as IL-1/IL-8, TNFR1/TNFR2, TWEAK, and relaxin signaling were found in triple-negative breast cancer. The dysregulated genes were clustered based on their mutation frequency which revealed nine mutated clusters, some of which were well characterized in cancer while others were less characterized. Each cluster was analyzed in detail which led to the identification of NLGN3, MAML2, TTN, SYNE1, ANK2 as candidate genes in BRCA. They are central hubs in the protein–protein-interaction network, indicating their important regulatory roles. Experimentally, the Real-Time Quantitative Reverse Transcription PCR and western blot confirmed our computational predictions in cell lines. Further, immunohistochemistry corroborated the results in ~ 100 tissue samples. We could experimentally show that the NLGN3 & ANK2 have tumor-suppressor roles in BRCA as shown by cell viability assay, transwell migration, colony forming and wound healing assay. The cell viability and migration was found to be significantly reduced in MCF7 and MDA-MB-231 cell lines in which the selected genes were over-expressed as compared to control cell lines. The wound healing assay also demonstrated a significant decrease in wound closure at 12 h and 24 h time intervals in MCF7 & MDA-MB-231 cells. These findings established the tumor suppressor roles of NLGN3 & ANK2 in BRCA. This will have important ramifications for the therapeutics discovery against BRCA.

## Introduction

BRCA is the most common cancer in females. It has become the leading cause of cancer worldwide. As per the Cancer statistics 2022, about 51,400 new cases of ductal carcinoma in situ of the female breast will be diagnosed. The new breast cancer cases rises to 297,790 and 2800 in female and male respectively as per the cancer statistics 2023. For women, breast cancer, colorectal cancers and lung cancer account for 52% of all new diagnoses, among which breast cancer alone accounts for 31% of female cancers ^1,2^. The late detection and emergence of resistance to existing therapy pose a significant challenge in the management of BRCA ^3^. Moreover, in-spite of recent advances in the understanding of the disease and the development of new therapies, the five-year survival rate is still unsatisfactory ^1,2^. Therefore, new targets are required for early detection and treatment to improve overall survival. In the present work, an in-depth analysis of RNA-sequencing data obtained from the cancer genome atlas (TCGA) for breast invasive carcinoma is reported. The dysregulated genes in three molecular classes of BRCA (ER/PR+/HER-2−; ER/PR−/HER-2+; triple negative) in the early and late stages have been identified. Further, different clusters of genes in various classes were investigated for their involvement in the biological processes. The subnetworks were found to contain frequently mutated genes in BRCA patients which can serve as therapeutic targets as well as biomarkers. The identified genes were further analyzed for their mutational patterns and effect on the overall survival of patients. The findings were finally validated by qRT-PCR, western blot in cell lines, and immunohistochemistry using ~ 100 tissue samples. The tumor suppressor role was demonstrated by cell viability assay, transwell migration, colony-forming and wound healing assay in MCF7, MDA-MB-231 & MCF10A cell lines.

## Results

### Differential expression analysis

The differential expression analysis was done separately for each of the five classes viz. early stage ER/PR+/HER-2−, late stage ER/PR+/HER-2−, Early stage ER/PR−/HER-2+, early stage triple-negative breast cancer (TNBC), and late stage TNBC using tumor and normal adjacent tissue (NAT) samples. The number of upregulated/downregulated genes in each class is reported in Table 1 and supplementary tables S1a–e. There were many dysregulated genes that were common among classes (supplementary fig. S1a, S1b, supplementary table S2a) whereas some were class and stage-specific (supplementary table S2b). Interestingly, some of the genes had inverse expression i.e. were upregulated in one class and downregulated in another (Table 2, (supplementary fig. S1c, S1d, supplementary table S2c). These observations indicate some common and stage-specific mechanisms underlie the pathology of different molecular classes of BRCA. The biological pathways were analyzed in each class (supplementary table S3a, S3b, S3c, S3d, S3e) and significant pathways have been discussed.

**Table 1:** Summary of dysregulated genes in early and late stage in each of the three molecular classes. The table shows significantly dysregulated genes with a log2 fold change greater than or less than 1. In early stage, there was a greater number of dysregulated genes as compared to late stage. The log2 fold change varied from 1-13 in early stages and 1-20 in late stages. In the case of late-stage ER/PR-/HER-2+, the analysis could not be done as there was no sample of NAT to compare with. The larger number of genes were found to be downregulated in all classes.

	Molecular class	No. of genes	Upregulated	Log_2__fold change range	Downregulated	Log_2__fold change range
			(Log_2__fold change >1; p-value < 0.05)		(Log_2__fold change <-1; p-value < 0.05)	
Early stage	ER/PR+/HER-2−	13,507	2274	1–9	3511	1–9
	ER/PR−/HER-2+	7754	503	1–13	5420	1–11
	TNBC	9403	1631	1–13	4507	1–11
Late stage	ER/PR+/HER-2−	10,071	1997	1–18	3866	1–13
	ER/PR−/HER-2+	No NAT sample				
	TNBC	3488	58	1–10.9	2859	1–20

**Table 2:** The genes showed inverse expression pattern among different classes in early and late stage. The genes which showed upregulation in one class were found to be downregulated in other class and vice-versa. The table indicates the number of such genes.

Early stage		Late stage	
Molecular class	Number of genes	Molecular class	Number of genes
Downregulated in ER/PR+/HER-2− & upregulated in TNBC	38	Downregulated in ER/PR+/HER-2− & upregulated in TNBC	2
Downregulated in ER/PR+/HER-2− & upregulated in ER/PR-/HER-2+	4	Upregulated in ER/PR+/HER-2− & downregulated in TNBC	155
Upregulated in ER/PR+/HER-2− & downregulated in TNBC	278		
Upregulated in ER/PR+/HER-2− & downregulated in ER/PR-/HER-2+	194		
Upregulated in TNBC & downregulated in ER/PR−/HER-2+	77		
Downregulated in TNBC & upregulated in ER/PR−/HER-2+	10		

### Mutated subnetworks of genes

The differentially expressed genes were clustered into subnetworks based on PPIs information and the number of SNPs they harbored, using hotnet2 algorithm ^4^. A total of nine significantly mutated clusters were found in different molecular classes (Table 3) containing hot (frequently mutated) and cold (less mutated) genes. The cold genes are implicated because of their interaction with hot genes (guilt-by-association). Pathway analysis indicated that some of them have a role in well-known cancer signaling pathways while others are less characterized. For each of the categories, different clusters were obtained that are discussed class-wise.

**Table 3:** Summary of results obtained after hotnet2 analysis. For each subnetwork, the table shows the minimum K for which subnetworks of size ≥ K are significant (p-value).

S.No.	Components	K	p-value
1	MYRIP, AMH, MYO7A, DDR2, PCSK5, PTPRM, CDH23, CDH1	8	0.01
2	SYT2, SYT9, SYT6, NLGN1, NLGN3, SYT13, NRXN1	7	0
3	PLIN2, APOB, LIPE, PLIN1, SEC61A2, STAR	6	0.01
4	MAML2, DLL4, NOTCH4, NOTCH2, DTX1, JAG1	6	0.032
5	VCAN, DNAH6, FBN1, EFEMP2, SELP, MFAP5	6	0.032
6	UNC5B, SYNE1, NEO1, NTN4, TTC3, TTN, MYOM1, MYOM2	8	0.01
7	VCAN, DNAH6, FBN1, ADAMTSL5, MFAP4, SELP, MFAP5	7	0
8	CHL1, CEP120, DMD, NFASC, GALK2, ANK1, ANK2, EHD2, CCDC18, L1CAM, NBPF1, TNS1, AHNAK, NBPF3	10	0.01
9	DAB1, APLP1, APOB, LRP2, CKLF, PCDHA6, PCDHA3, RELN, SLC12A4, ZDHHC11, CUBN	10	0.01

#### Early stage ER/PR+/HER-2−

**Figure 1:**
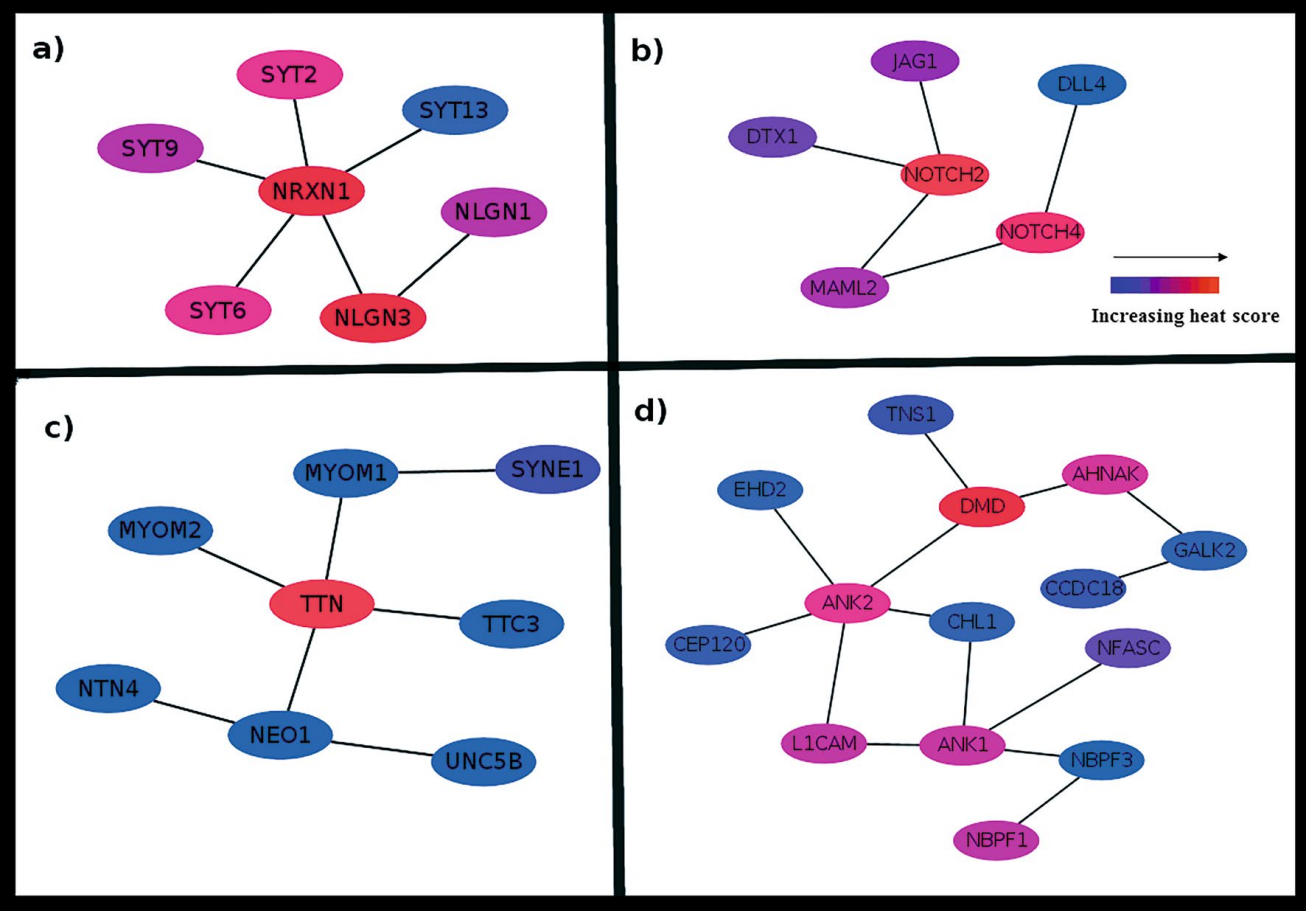
Subnetwork for (a) Early stage ER/PR+/HER-2-. Seven dysregulated genes, coloured by the increasing mutation frequency (blue to red). NLGN3 and NRXN1 are frequently mutated while SYT13 is less mutated. The pathway analysis showed enrichment of TR/RXR activation pathway which activates PI3K pathway and regulates the biological process involved in cell survival and cell growth. (b) Early stage ER/PR-/HER-2+ containing six dysregulated genes. The NOTCH2 and NOTCH4 are highly mutated. The subnetwork may be involved in Notch signaling pathway, Th1 and Th2 activation pathway and epithelial-mesenchymal transition pathway. (c) Early stage TNBC. Contains eight dysregulated genes among which TTN is highly mutated which is followed by SYNE1. Netrin signaling, RhoA signaling, and axonal guidance signaling pathways may be affected. (d) Late stage TNBC. Contains fourteen dysregulated genes. DMD2 and ANK2 are significantly enriched in mutations. The subnetwork may regulate galactose degradation I pathway, colanic acid building blocks biosynthesis and nNOS signaling pathway.

Three clusters were obtained, out of which one is significantly mutated (p-value < 0.05) containing seven genes viz. SYT2, SYT6, SYT9, SYT13, NRXN1, NLGN1 and NLGL3 (Fig. 1a). Among them, 2 were upregulated and 5 were downregulated with average log_2_ fold change (FC) as 2.21 Table 4 The pathway analysis indicated that the cluster may be involved in TR/RXR activation pathway (supplementary fig. S2), which activates PI3K pathway and is involved in cell survival and growth ^5^.

**Table 4:** Summary of each component of the subnetwork. The table is showing heat score and fold change of each component. The status and mRNA expression of the components in expression atlas and Oncomine is also shown.

Component	Heat score	Log_2_ fold change	Expression (RNA-seq data analysis)*	Expression Atlas mRNA	Oncomine mRNA expression	Canonical pathways & p-value	
SYT2	0.0079	− 1.03	Down	Down	Down	TR/RXR activation pathway	3.43E−03
SYT9	0.0059	2.09	Up	Down	Up		
SYT6	0.0079	− 1.44	Down	Down	Down		
NLGN1	0.0059	− 1.93	Down	–	Down		
NLGN3	0.0177	− 1.06	Down	Down	Down		
SYT13	0.002	5.67	Up	Down	Up		
NRXN1	0.0167	− 2.22	Down	Down	Down		

* Log_2_FC>1 or Log_2_FC<-1, p-value < 0.05.

#### Early stage ER/PR−/HER-2+

Two significant clusters were identified and the most significant one had 6 downregulated genes viz*.* JAG1, NOTCH2, DTX1, MAML2, NOTCH4, and DLL4 (average log_2_ FC = 1.60, Fig. 1b, Table 5). The cluster was found to be involved in Notch signaling, Th1 and Th2 activation, and regulation of epithelial-mesenchymal transition (EMT) pathways (supplementary fig. S3a, S3b, S3c).

**Table 5:** Summary of each component of the subnetwork. The heat score value of each component is calculated based on the mutation frequency. The subnetwork is found to be involved in pathways e.g. Notch signaling pathway, Th1 and Th2 activation pathway and regulation of epithelial-mesenchymal transition pathway (EMT).

Component	Heat score	Log_2_ Fold change	Expression (RNA-seq data analysis)*	Expression Atlas mRNA	Oncomine mRNA expression	Canonical pathways & p-value	
MAML2	0.0108	− 2.15	Down	Down	Down	Notch signalling pathway, Th1 and Th2 activation pathway and regulation of epithelial-mesenchymal transition pathway	2.88e−17, 1.00e−07 and 1.82E-05
DLL4	0.002	− 1.02	Down	Down	Down		
NOTCH4	0.0177	− 1.98	Down	Down	Down		
NOTCH2	0.0294	− 1.49	Down	Down	Down		
DTX1	0.0079	− 1.77	Down	Down	Down		
JAG1	0.0098	− 1.21	Down	Down	Down		

* Log_2_FC>1 or Log_2_FC<-1, p-value < 0.05.

#### Early stage TNBC

Two mutated subnetworks were identified and the most mutated composed of 8 genes viz. TTN, TTC3, MYOM1, MYOM2, SYNE1, NEO1, NTN4 and UNC5B (average log_2_ FC = 2.17). All genes in the subnetwork were significantly downregulated except UNC5B. (Fig. 1c, Table 6). TTN and SYNE1 are known driver genes in BRCA and were significantly mutated as compared to others ^6^. Pathway analysis showed enrichment of netrin signaling, RhoA signaling, and axonal guidance signaling pathways (supplementary fig. S4a, S4b, S4c). Axonal guidance signaling play a crucial mechanism in tumor suppression and oncogenesis and can be promising targets for therapeutics ^7,8^.

**Table 6:** Summary of each component of the subnetwork in early stage TNBC class. The table also depicts status and mRNA expression of each component in expression atlas and Oncomine.

Component	Heat score	Log_2_ Fold change	Expression (RNA-seq data analysis)*	Expression Atlas mRNA	Oncomine mRNA expression	Canonical pathways & p-value	
UNC5B	0.0088	1.58	Up	Up	Up	Netrin signalling, RhoA signalling, and axonal guidance signaling pathways	2.61e−02, 4.85e−02 and 0.0133
SYNE1	0.0558	− 1.68	Down	Down	Down		
NEO1	0.0088	− 1.11	Down	Down	Up		
NTN4	0.0049	− 2.87	Down	Down	Down		
TTC3	0.0127	− 1.17	Down	Down	Up		
TTN	0.2008	− 2.9	Down	Up	Down		
MYOM1	0.0078	− 4.15	Down	Down	Down		
MYOM2	0.0147	− 1.9	Down	Down	Down		

* Log_2_FC>1 or Log_2_FC<-1, p-value < 0.05.

#### Late stage TNBC

Two mutated clusters were found and most significant had 14 genes (Fig. 1d, Table 7) (average log_2_ FC 2.59). It is important to note that TNS1, DMD, ANK2 ^6^, and AHNAK ^9^ are known driver genes in BRCA. The cluster was found to be involved in galactose degradation, colanic acid building blocks biosynthesis, and nNOS signaling pathway (supplementary fig. S5a, S5b, S5c). It has been reported that the knockdown of galactokinase controls the growth of hepatoma cells in vitro ^10^ and galactose metabolism pathways are dysregulated in breast cancer ^11^.

**Table 7:** Summary of each component of the subnetwork in late stage TNBC. The table also represents status and mRNA expression of each component in expression atlas and Oncomine. The subnetwork appears to be involved in pathways as galactose degradation I, colanic acid building blocks biosynthesis and nNOS signaling pathway.

Component	Heat score	Log_2_ Fold change	Expression (RNA-seq data analysis)*	Expression Atlas mRNA	Oncomine mRNA expression	Canonical pathways & p-value	
CCDC18	0.0099	2.03	Up	Up	Up	Galactose degradation I pathway, colanic acid building blocks biosynthesis and nNOS signaling pathway	3.55e−03 9.91e−03 and 2.81E−02
CEP120	0.0089	− 2.82	Down	Down	Down		
NBPF3	0.003	− 1.85	Down	Up	Up		
AHNAK	0.0277	− 1.33	Down	Down	Down		
NBPF1	0.0238	− 2.89	Down	Up	Down		
EHD2	0.0059	− 1.51	Down	Down	Down		
NFASC	0.0149	− 2.54	Down	Down	Up		
CHL1	0.0069	− 2.27	Down	Down	Down		
TNS1	0.0109	− 1.8	Down	Down	Up		
L1CAM	0.0248	− 2.85	Down	Down	Up		
GALK2	0.0059	− 3.21	Down	Down	Down		
DMD	0.0465	− 3.18	Down	Down	Down		
ANK2	0.0307	− 2.2	Down	Down	Down		
ANK1	0.0257	− 5.74	Down	Up	Down		

* Log_2_FC>1 or Log_2_FC<-1, p-value < 0.05.

The cluster from all classes contains total 35 genes. It was interesting to see many genes in the identified clusters which are not yet reported to be directly involved in BRCA. Initial analysis indicated that these genes have reported interactions with known cancer genes. Some of them are regulators of genes having known role in BRCA. Additionally, many of them were also found to be dysregulated across other cancers ^12^ (Supplementary fig. S6). Therefore, it is reasonable to assume that they may also play some role in BRCA. The genes were further examined for mutations e.g. inframe, truncating, missense or others using cBioPortal ^13^. A large number of mutations were found in TTN, SYNE1, ANK2, and NLGN3. In fact, few genes showed mutation frequency greater than BRCA1/BRCA2.

### Detailed mutational analysis of significant clusters

#### Early stage ER/PR+/HER-2−: NLGN3, NRNX1, NLGN1 cluster

**Figure 2:**
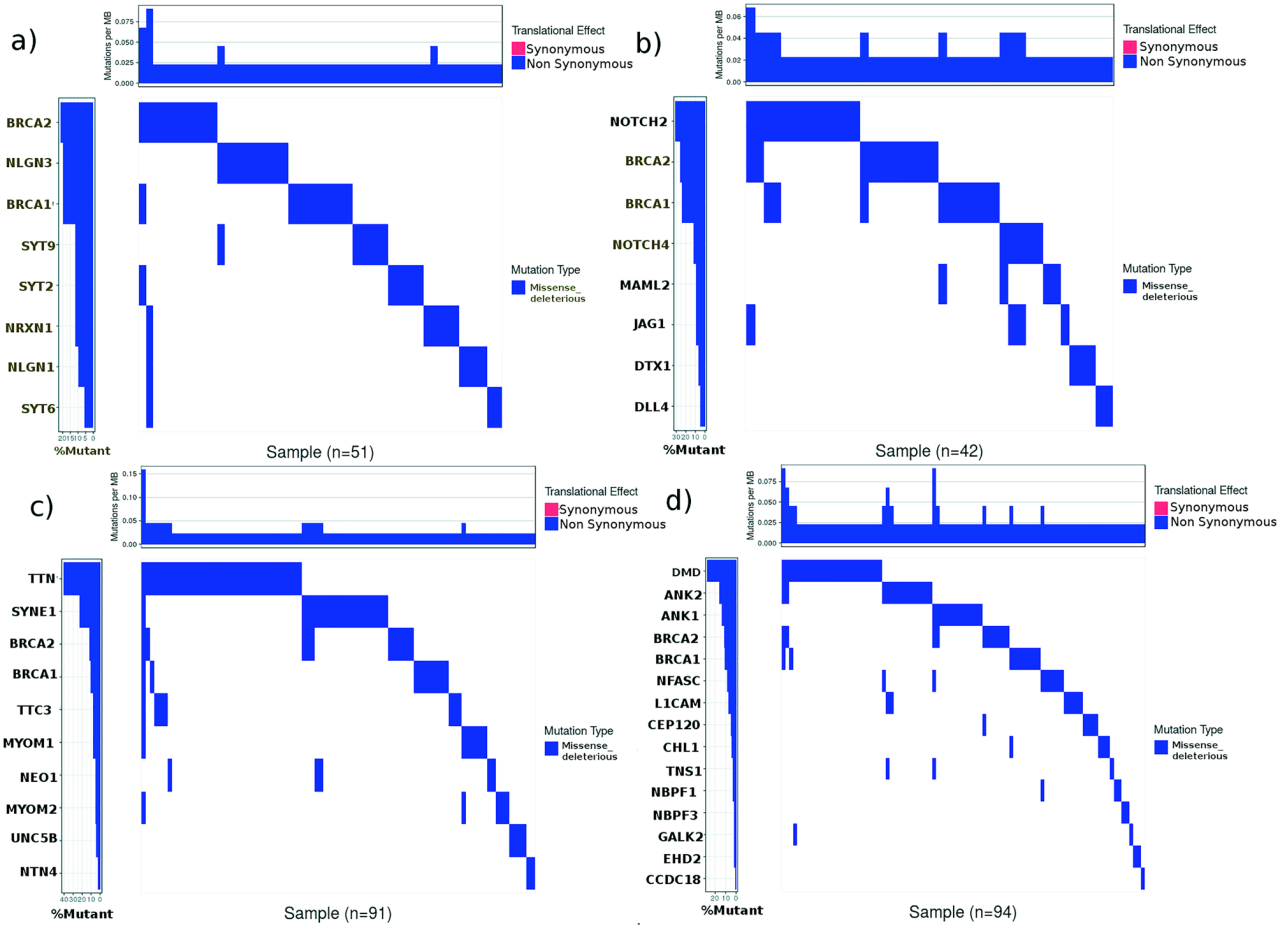
Mutational landscape of candidate genes showing deleterious functional impact in (a) 51 out of 60 samples, (b) 42 out of 59 samples (c) 91 out of 254 samples (d) 94 out of 164 samples. For each sample, the mutations per MB was calculated using number of deleterious mutations present. The mutation rate for a gene is based on deleterious missense mutation in number of samples. The mutation rate in NLGN3 is equal to BRCA1 (10 deleterious mutations). The mutation rate in TTN, SYNE1, ANK1 and ANK2 is greater than BRCA1 and BRCA2.

In NLGN1 and NLGN3, carboxylesterase domain contains the majority of the mutations whereas in NRXN1, laminin G domain contains the majority of mutations. The mutations in the laminin domain may contribute to EMT by affecting cell-to-cell adhesion by binding to integrin α6β4 (β4 subunit) expressed by epithelial cells. This promotes tumor cell survival, invasion, and migration via signaling through RAC and PI3K pathway ^14^. In other members (SYT2, SYT6, SYT9 and SYT13), C2 domain was affected. The C2 domains of SYT family are involved in exocytosis. The alterations of exocytic proteins are known to be associated with malignant transformations ^15^. The altered exocytic networks are involved in the acquisition of premetastatic traits ^16^. Therefore, the identification of such a cluster is no surprise. The details of the mutations in each gene are given in Table 8 and supplementary Table S4a. Such mutations which can impact protein function have been plotted against tumor samples (Fig. 2a). Among all genes in this subnetwork, the NLGN3 showed a higher mutation rate, equal to BRCA1.

**Table 8:** Mutational detail of the components of subnetwork showing total mutations (unique) across the four studies, TCGA; Cell 2015, TCGA; Nature 2012, TCGA; PanCancer Atlas and TCGA; Provisional using cbioportal. The cluster harbored several types of mutations as missense, fusion, splice, frame-shift, in-frame. The somatic mutation frequency of NLGN3 and NRXN1 was found to 1.0%.

Gene name	Total mutations	Mutation type	Mutation showing deleterious impact	Somatic mutation frequency (%)
SYT2	10	Missense, Fusion	6	0.6
SYT9	8	Missense, Fusion	6	0.3
SYT6	8	Missense, Splice, Frame_shift_deletion	3	0.4
NLGN1	9	Missense, Fusion, Nonsense	6	0.5
NLGN3	16	Missense, Splice, Frame_shift_deletion, Frame_shift_insertion	10	1
SYT13	1	Missense	0	0.1
NRXN1	22	Missense, Fusion, Frame_shift_deletion, Frame_shift_insertion	7	1
BRCA1	42	Missense, Splice, Nonsense, Frame_shift_deletion, Frame_shift_insertion, In_frame_deletion	10	1.7
BRCA2	50	Missense, Splice, Nonsense, Fusion, Frame_shift_deletion, Frame_shift_insertion, In_frame_deletion	11	1.9

##### NLGN3 as a candidate gene in early stage ER/PR+/HER-2−

**Figure 3:**
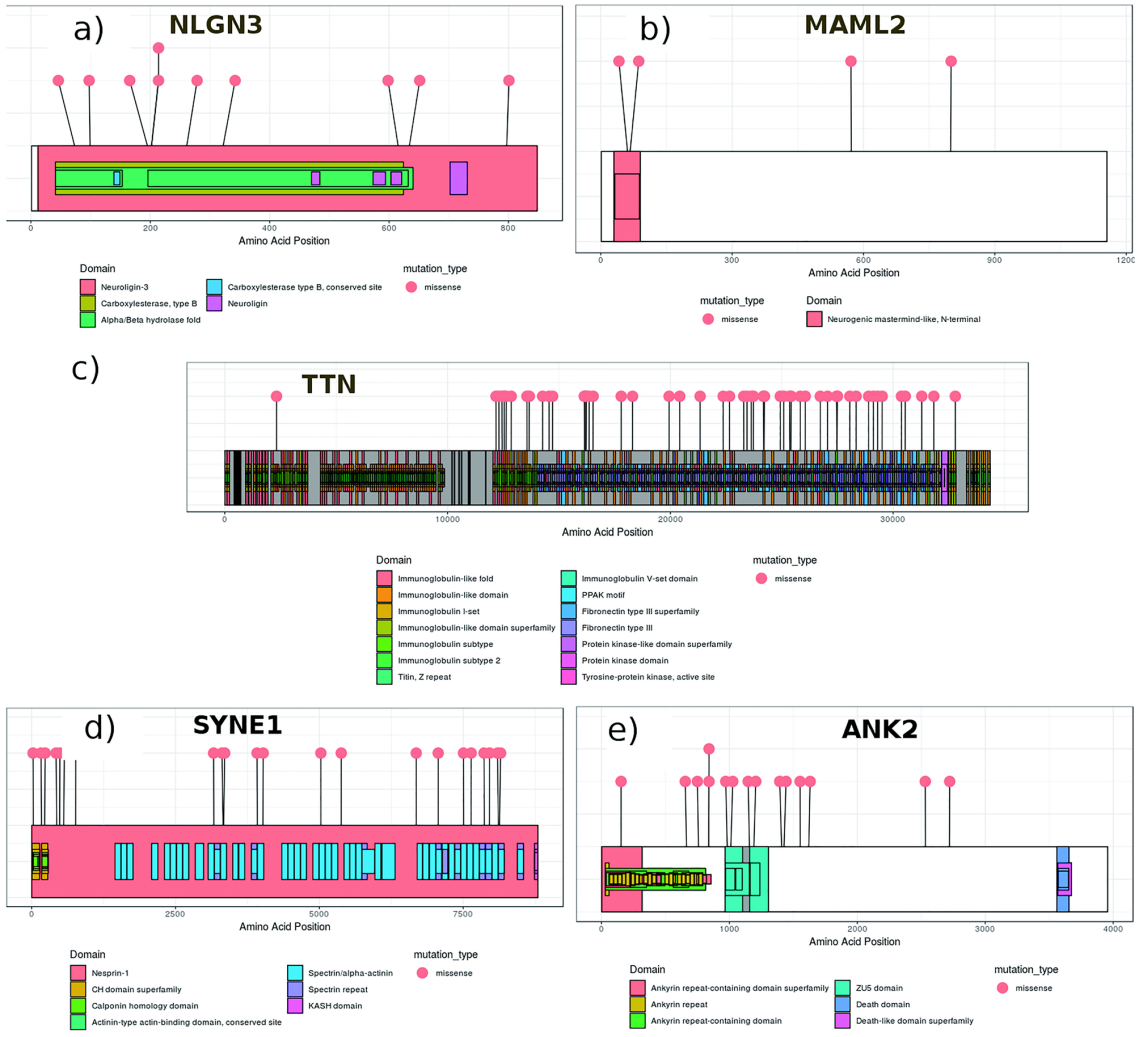
Lollipop plot representing missense mutations (deleterious) in functional domains of (a) NLGN3, (b) MAML2, (c) TTN, (d) SYNE1 and (e) ANK2. In NLGN3, carboxylesterase family, neurolgin-3 and alpha-beta hydrolase domain were significantly enriched in mutation. In MAML2, neurogenic mastermind like N-terminal domain harbored mutations as G61D and D67N. All functional domains in TTN were occupied with mutations. In SYNE1, the spectrin/alpha-actinin and spectrin-repeat domain got enriched and in ANK2, ZU5 domain got enriched.

The fact that the NLGN3 is dysregulated and highly mutated in a number of samples makes it an interesting candidate. NLGN3 is a protein-coding gene that is reported to be involved in Asperger’s syndrome and autism X-linked 1. The gene was found to be significantly downregulated (log_2__FC = − 1.06) (supplementary fig. S7a) and interacts with other genes viz. DLG4, GABRA1, OR1F12, CYFIP2, GABRA2, AGAP2, NLGN4X, UTP15, NLGN4Y, TMEM39A, TYK2, RECQL4, PIK3CB, PIK3CA, NLGN1 ^17^. Many of them are well-known cancer-associated genes. The NLGN3 was also found to have a role in cell–cell adhesion and cell differentiation ^18^. Additionally, it regulates GRIN2B ^19^, which is reported to play a significant role in cancer development and progression. In NLGN3, total of sixteen mutations were found in different domains; among them ten were predicted deleterious. Figure 3a shows that carboxylesterase family, neurolgin-3, and alpha–beta hydrolase domain got significantly enriched in mutations. Therefore, considering the above reasons, the gene NLGN3 can be a good candidate gene.

#### Early stage ER/PR-/HER-2+: NOTCH and interactors

NOTCH2 and NOTCH4 are well-known genes in BRCA and other cancers. Most of the mutations in NOTCH2 and NOTCH4 were found to be missense that are spread over different domains viz. EGF-like, calcium-binding EGF and NOTCH protein. A few truncating mutations also occurred in the EGF-like domain and domain of unknown function (DUF). The mutations in the EGF-like domain lead to the inactivation of NOTCH ^20^. Other members were found to be less mutated and clustered due to their association with NOTCH. The details of different mutations in each of the gene is given in Table 9 and supplementary table S4b. MAML2 harbored total 12 mutations, out of which, 4 were predicted to be deleterious. Figure 2b shows the percent mutation rate for each member. Although percent mutation rate for MAML2 is less, it interacts with NOTCH1 and NOTCH2. Its precise role in BRCA pathogenesis is not yet known.

**Table 9:** Mutational detail of the NOTCH gene and interactors representing total unique mutations across the four studies, TCGA; Cell 2015, TCGA; Nature 2012, TCGA; PanCancer Atlas and TCGA; Provisional. The components harbored several types of mutations as missense, fusion, splice, frame-shift, in-frame as shown in the table.

Gene name	Total mutations	Mutation type	Mutation showing deleterious impact	Somatic mutation frequency (%)
MAML2	12	Missense, Fusion, Frame_shift_deletion, Frame_shift_insertion	4	0.6
DLL4	3	Missense	2	0.2
NOTCH4	21	Missense, Frame_shift_deletion, In_frame_deletion, In_frame_insertion	5	0.9
NOTCH2	46	Missense, Fusion, Non-sense, Frame_shift_deletion, Frame_shift_insertion	13	2.2
DTX1	7	Missense, Fusion, Nonsense	3	0.4
JAG1	14	Missense, Splice, Nonsense, Frame_shift_deletion	4	0.7

##### MAML2 as a candidate gene in early stage ER/PR−/HER-2+

MAML2, a mastermind like transcriptional coactivator 2, encodes gene of mastermind-like family of proteins. The gene is reported to be involved in Notch signaling and pre-NOTCH expression and processing pathways. The N-terminal domain binds CREBBP/CBP ^21^ and CDK8 ^22^. It regulates various genes viz*.* ID2, NR4A2, FOS, STC1, NR4A1, CREM, ATF3, MAF, CTH, KRT17, PTN, CRYAB, THBS1, CGB3, HAS2 ^17^, many of them are known for their association to BRCA pathogenesis ^23–32^. In BRCA, the neurogenic mastermind like N-terminal domain in MAML2 was found to be affected by many deleterious mutations (Fig. 3b). It was interesting to find MAML2 to be downregulated in early stage ER/PR−/HER-2+ samples (log_2__FC = − 2.14) (supplementary fig. S7b). Being co-activator of NOTCH protein, which is having a dual role as an oncogene and tumor-suppressor ^33^, MAML2 might be having tumor-suppressor role in BRCA or might be leading to oncogenesis through some other pathways, but the molecular mechanism is not yet clear. Therefore, it would be interesting to understand the role of MAML2 downregulation in BRCA samples.

#### Early stage TNBC: TTN and interactors

The TTN was found to be the most mutated gene followed by SYNE1. The other members of networks were less mutated with somatic mutation frequency (SMF) <  = 1%. In TTN, the total mutations were 438, (366-missense, 72-truncating and 4-inframe). In SYNE1, the total mutations were found to be 105, (79-missense, 20-truncating, Table 10 and supplementary table S4c). The percent mutation rate for TTN and SYNE1 is significantly high which may make them ideal candidate genes in BRCA (Fig. 2c).

**Table 10:** Mutational detail of TTN gene and its interactors in early stage TNBC. The data represents the total unique mutations across the carcinoma samples across four studies (TCGA; Cell 2015, TCGA; Nature 2012, TCGA; PanCancer Atlas and TCGA; Provisional). The cluster harbored several types of mutations as missense, nonsense, fusion, splice, frame-shift, in-frame. The somatic mutation frequency of TTN was found to be 14.4% which is very high as compared to known genes (BRCA1 and BRCA2). Also, SYNE1 showed somatic mutation frequency as 4.4% which is even much greater than BRCA1 (1.7%) and BRCA2 (1.8%).

Gene name	Total mutations	Mutation type	Mutation showing deleterious impact	Somatic mutation frequency (%)
UNC5B	9	Missense, Splice, Frame_shift_deletion	4	0.3
SYNE1	105	Missense, Nonsense, Frame_shift_deletion, Splice, Fusion	22	4.4
NEO1	9	Missense, Splice, Fusion	5	0.6
NTN4	3	Missense, Nonsense	2	0.1
TTC3	17	Missense, Fusion, Nonsense, Frame_shift_deletion	7	0.8
TTN	438	Missense, Splice, Nonsense, Frame_shift_deletion, In_frame_deletion, In_frame_insertion, Frame_shift_insertion	55	14.4
MYOM1	12	Missense, Nonsense, Frame_shift_deletion	7	0.7
MYOM2	19	Missense, Nonsense, Frame_shift_deletion	5	1
BRCA1	42	Missense, Splice, Nonsense, Frame_shift_deletion, Frame_shift_insertion, In_frame_deletion	10	1.7
BRCA2	50	Missense, Splice, Non-sense, Fusion, Frame_shift_deletion, Frame_shift_insertion, In_frame_deletion	11	1.9

##### TTN as a candidate gene in early stage TNBC

TTN is a protein-coding gene associated with myopathy and muscular dystrophy. The gene contains immunoglobulin, kinase, Mex5, N2A, and N2b spring domain. It was found to be significantly downregulated (log_2_ FC = − 2.9) in early stage TNBC (supplementary fig. S7c). The immunoglobulin and kinase domains were found to be majorly affected by missense mutations. A very large number of mutations (55 of 438) in TTN were predicted as deleterious, which makes it noteworthy. The gene is involved in actin cytoskeleton signaling, adrenomedullin signaling, integrin signaling, protein kinase A signaling and RhoA signaling. It regulates the function of FHL2, ANKRD2, CRYAB, FHL1, PLN, Calm1, ATP2A2, TRIM63, CABLES1 genes ^34–39^ which might be involved in the progression of BRCA. The TTN plays role in cell growth regulation and maintain structural integrity of the cell. Its molecular function is associated with ankyrin binding and protein kinase activity ^40^. Figure 3c shows immunoglobin-like family and fibronectin domains to be significantly enriched in mutations.

##### SYNE1 as candidate gene in early stage TNBC

It is protein-coding gene that encodes spectrin repeat containing protein. The gene is associated with muscular dystrophy and contains KASH (Nuclear envelop localization), calponin homology (CH), and spectrin repeat domain. It is involved in processes such as cell death, migration, differentiation, and proliferation ^41^. The gene regulates glutamate receptor and binds DLGAP1, IFT57, SUN1, TNIK, NDEL1, EMD, LMNA, SYNE1, DISC1, HEY1, DLG4, SUN3, AGAP2, ATP1B4, TUBB2B ^17^. The gene is significantly downregulated ^42^ (log_2_ FC = − 1.7) and highly mutated gene in TNBC (supplementary fig. S7d). It was found to have 22 deleterious mutations out of 79 missense mutations affecting CH and spectrin repeat domain (Fig. 3d). The spectrin-based domain protects epithelial cells from mechanical stress and is involved in the homeostasis of water and salt. The mutation in the spectrin domain may lead to its loss of function which results in defective TGF-B signaling and cell cycle deregulation. The loss of this domain is a marker of metastatic cancer cells ^43^. The plot shows spectrin/alpha-actinin and spectrin repeat domain got significantly enriched in mutations.

#### Late stage TNBC: DMD, ANK1, ANK2 and interactors

DMD and ANK2 were found to be highly mutated. DMD is a well-known gene ^44^ but there are no reports for the role of ANK2 in BRCA. ANK2 (SMF = 2.0%) directly interacts with DMD (SMF = 3.6%) and most mutations in ANK2 and DMD were found to be missense. ANK1 and AHNAK had SMF as 1.5% and 1.4% respectively and other members of the cluster were less mutated (SMF < 1%). Among all, six were well-known in BRCA while the rest (NBPF3, ANK1, NFASC, CHL1, ANK2, CEP120, GAK2, and CCDC18) are not reported yet. Out of these, we found ANK2 as the most mutated (Table 11 and supplementary table S4d) with a mutation rate higher than BRCA/BRCA2. (Fig. 2d).

**Table 11:** Mutational detail of DMD, ANK1, ANK2 and interactors in late stage TNBC. The data represents the total unique mutations across the carcinoma samples across four studies (TCGA; Cell 2015, TCGA; Nature 2012, TCGA; PanCancer Atlas and TCGA; Provisional). The cluster harbored several types of mutations as missense, nonsense, fusion, splice, frame-shift, in-frame. Among the network, ANK2 harbored greater rate of somatic mutation frequency i.e. 2.0%, even greater than BRCA1 and BRCA2.

Gene name	Total mutations	Mutation type	Mutation showing deleterious impact	Somatic mutation frequency (%)
CCDC18	12	Missense, Nonsense, Frame_shift_deletion, Splice, Fusion	1	0.5
CEP120	11	Missense, Nonsense, In_frame_deletion	5	0.7
NBPF3	6	Missense, Splice	3	0.2
AHNAK	47	Missense, Fusion, Frame_shift_deletion	0	1.4
NBPF1	27	Missense, Splice, Frame_shift_deletion	3	0.9
EHD2	6	Missense, Nonsense, Frame_shift_deletion	2	0.4
NFASC	21	Missense, Frame_shift_insertion, Splice, Frame_shift_deletion, Fusion	8	1
CHL1	8	Missense, Non-sense	6	0.4
TNS1	13	Missense, Frame_shift_insertion, Frame_shift_deletion, In_frame_deletion	3	0.7
L1CAM	12	Missense, Frame_shift_deletion	7	0.8
GALK2	6	Missense, Fusion	2	0.3
DMD	74	Missense, Nonsense, Frame_shift_deletion, Frame_shift_insertion, Splice	29	3.6
ANK2	40	Missense, Splice, Non-sense, Frame_shift_deletion	15	2
ANK1	37	Missense, Fusion, Frame_shift_deletion, Frame_shift_insertion, Nonsense, Splice	18	1.5
BRCA1	42	Missense, Splice, Nonsense, Frame_shift_deletion, Frame_shift_insertion, In_frame_deletion	10	1.7
BRCA2	50	Missense, Splice, Non-sense, Fusion, Frame_shift_deletion, Frame_shift_insertion, In_frame_deletion	11	1.9

##### ANK2 as candidate gene in late stage TNBC

It is protein-coding gene, encoding ankyrin-family of proteins. The binding site in ankyrins facilitates binding of integral membrane proteins with spectrin-actin based membrane cytoskeletal. This linkage maintains the integrity of plasma membrane and anchors specific ion channels. They play role in cellular activities e.g. motility and proliferation ^45^. The gene is significantly downregulated (log_2_ FC = − 2.2) (supplementary fig. S7e) in TNBC harboring 40 mutations, out of which 32 were missense. The ankyrin repeat domain and ZU5 domains were mainly affected by missense mutations (Fig. 3e). Ankyrin repeat domains are present in diverse proteins and act as platform for interactions with other proteins. The ankyrin repeat domain binds to miRNA, causing a loss in their function and drives proliferation in renal cancer cells ^46^. Additionally, the overexpression of ankyrin repeat domain is related to the drug resistance in lung cancer ^47^. The ZU5 domain is involved in the induction of apoptosis and binds to the NRAGE domain of UNC5H. The ANK2 regulates ITPR1, ITPR, RYR2, MAPK1, ERK1/2, SPTBN1, focal adhesion kinase, SCN8A and ANK3. The ANK2 is involved in molecular functions such as protein kinase binding, ATPase binding and structural constituent of cytoskeletal. ().

### Experimental validation

Validation of predictions is a quintessential part of scientific research. In the current studies to validate the predictions, one gene from each of the subnetworks was picked, thus four genes viz. NLGN3, ANK2, MAML2 and SYNE1 were selected. The transcript level of selected genes in different breast cancer cell lines MCF7, T47D, MDA-MB-231 was determined.

#### qRT-PCR analysis

**Figure 4:**
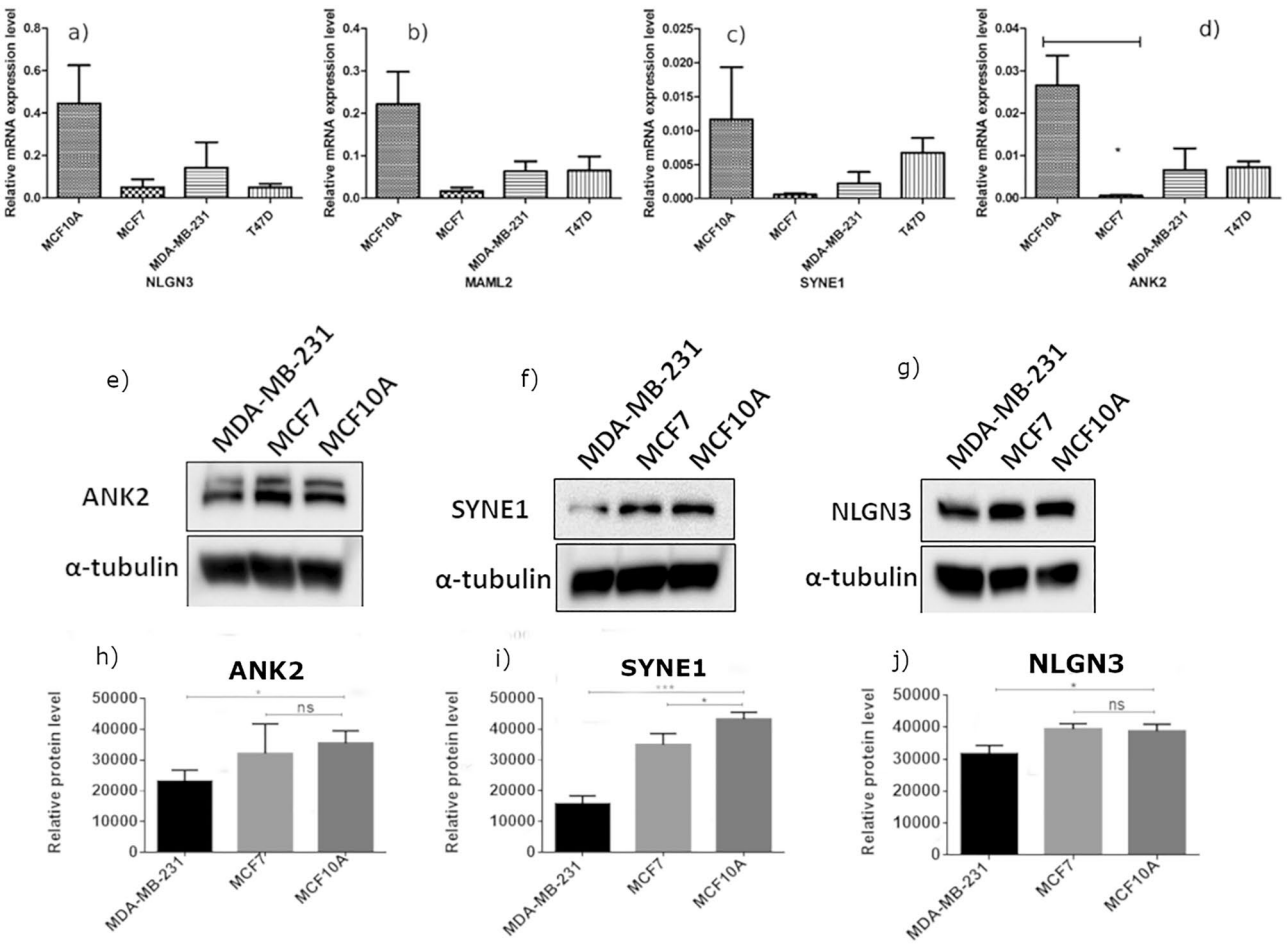
Real-Time PCR and western blot analysis of selected genes viz. NLGN3, MAML2, SYNE1 and ANK2. Figure shows relative mRNA expression level of a) NLGN3 b) MAML2 c) SYNE1 d) ANK2 in MCF10A, MCF7, MDA-MBA-231 and T47D cell lines. The GAPDH was used as control. Western blot illustrates the expression level of e) ANK2, f) SYNE1 and g) NLGN3 in different breast cancer cell lines (MDA-MB-231 and MCF7) and normal breast epithelial cell line (MCF10A). The blots were quantified by densitometry and subjected to statistical analysis and figures are shown in h), i) and j) respectively for ANK2, SYNE1 and NLGN3. Overall data are represented as mean ± SD. Student’s t-test was used for the statistical analysis, n = 3 (***P ≤ 0.001, *P ≤ 0.05, Significant). Ns: no significant difference.

The amplification of GAPDH was completed earlier than other candidate genes as revealed by CT value, indicating that the genes are down-regulated in cancer cell lines. The expression of NLGN3 was 0.12, 0.15, 0.30-fold downregulated in MCF7, T47D, and MDA-MB-231 respectively. The expression of MAML2 was 0.16, 0.33, and 0.29-fold (downregulated) in MCF7, T47D, and MDA-MB-231 respectively. The expression of SYNE1 was 0.08 and 0.17-fold (downregulated) in MCF7 and MDA-MB-231 respectively and 1.44-fold upregulated in T47D. The expression of ANK2 was 0.06, 0.27and 0.24-fold (downregulated) in MCF7 and MDA-MB-231 respectively (Fig. 4a–d).

#### Western blot analysis

The blot images of ANK2, SYNE1 and NLGN3 are represented in Fig. 4e–g respectively. The unpaired student’s t-test showed the decrease in protein expression of ANK2, SYNE1 and NLGN3 was significant in the cancer cell lines MDA-MB-231 (p-value < 0.05) when compared with control MCF10A and decrease in protein expression of SYNE1 was significant in MCF7 breast cancer cell line (Fig. 4h–j). The full length western blots are given in Supplementary Information file.

#### Immunohistochemistry

**Figure 5:**
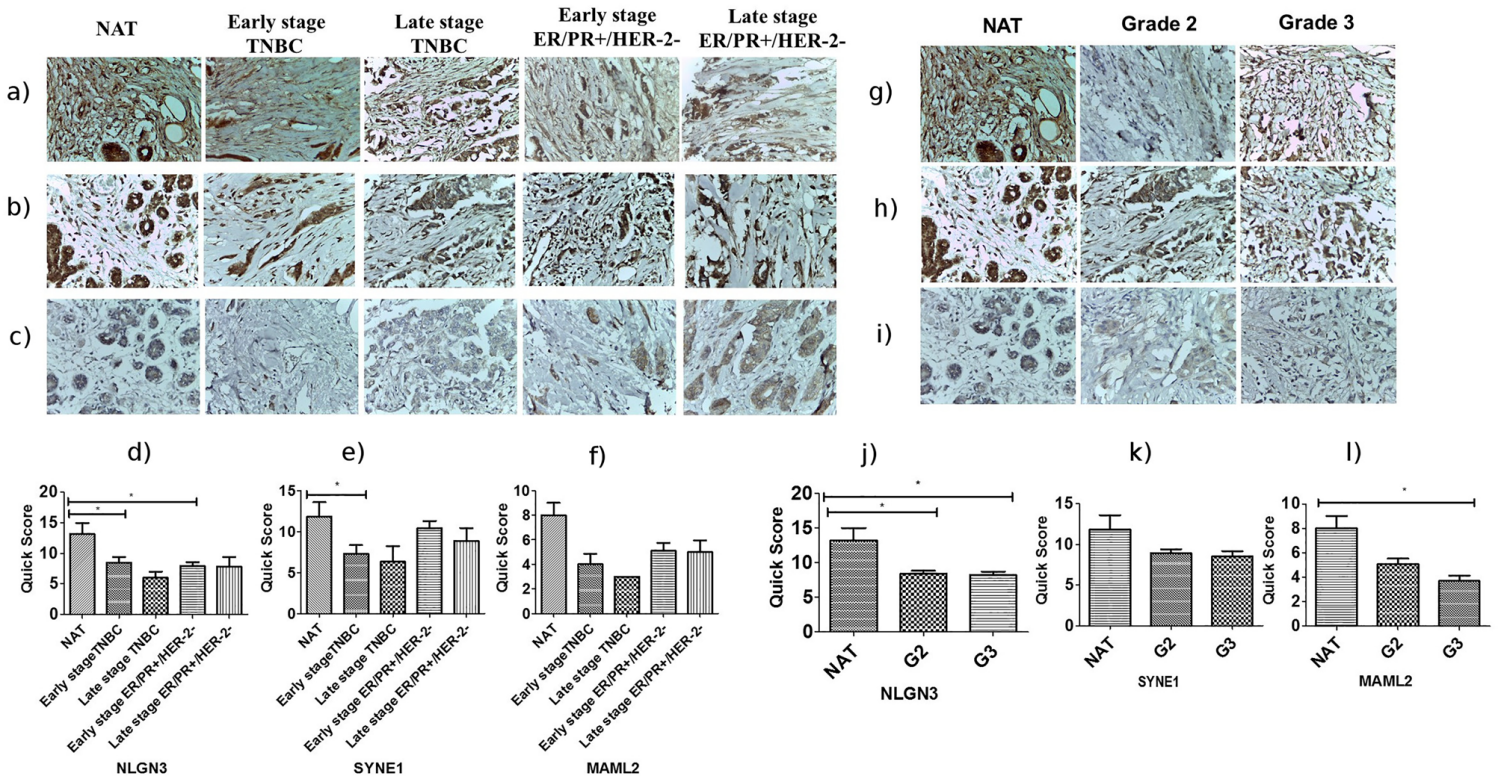
Figure showing the immunohistochemically stained TMA sections for a) NLGN3 b) SYNE1 and c) MAML2 in NAT, early stage TNBC, late stage TNBC, early stage ER/PR+/HER-2- and late stage ER/PR+/HER-2-. d) NLGN3 expression is significantly decreased in early stage ER/PR+/HER-2- (p-value=0.0162) and early stage TNBC (p-value=0.0328). e) SYNE1 expression is significantly downregulated in early stage TNBC (pvalue=0.0410). f) Downregulated expression of MAML2 was found in all 4 categories but was not statistically significant. Right panel shows the immunohistochemically stained TMA sections for g) NLGN3 h) SYNE1 and i) MAML2 in NAT, grade 2 and grade 3 tumor. j) The expression of NLGN3 is significantly decreased in grade 2 (pvalue=0.0137) and grade 3 (p-value=0.0123) compared to NAT. k) There was a decrease in SYNE1 expression in grade 2 and 3 tumor samples but it was statistically insignificant. l) MAML2 expression is significantly decreased in grade 3 (p-value=0.017). Data are represented as mean ± SD. Student’s t-test was used for the statistical analyses, n = 3 (*P < 0.05).

**NLGN3:** The mean expression of neurolgin3 was found to be 7.91, 7.86, 8.5, 6.0 and 13.2 in early stage ER/PR+/HER-2−, late stage ER/PR+/HER-2−, early stage TNBC, late stage TNBC and NATs samples respectively. It reveals that the neurolgin3 is downregulated in each of the four categories. The Mann–Whitney test revealed significant downregulation of NLGN3 in early stage ER/PR+/HER-2− (p-value = 0.0162) and early stage TNBC (p-value = 0.0328) (Fig. 5a, d). The grade-wise comparison showed that the NLGN3 is significantly downregulated in grade 2 (p-value = 0.0137) and grade 3 (p-value = 0.0123) (Fig. 5g, j).

**SYNE1:** The tissue expression of SYNE1 is decreased in each of the four categories. The mean expression was found to be 10.41, 8.86, 7.25, 6.33 and 11.83 in early stage ER/PR+/HER-2−, late stage ER/PR+/HER-2−, early stage TNBC, late stage TNBC and NATs respectively. The Mann–Whitney test revealed a significant decrease in SYNE1 expression in early stage TNBC (p-value = 0.0410) (Fig. 5b, e). The tissue expression of SYNE1 is also decreased in grade 2 and 3 tumors but was not statistically significant (Fig. 5h, k).

**MAML2:** The mean expression of MAML2 was found to be 5.11, 5, 4, 3, 8 in early stage ER/PR+/HER-2−, late stage ER/PR + /HER-2-, early stage TNBC, late stage TNBC and NATs respectively. The tissue expression of MAML2 is downregulated in early stage ER/PR+/HER-2− and early stage TNBC but was not statistically significant (Fig. 5c, f). The grade-wise analysis showed that the MAML2 is significantly downregulated in grade 3 (p-value = 0.017). (Fig. 5i, l).

#### Elucidation of tumor suppressor role of ANK2 and NLGN3 using cell viability, colony forming, transwell migration and wound healing assay

**Figure 6:**
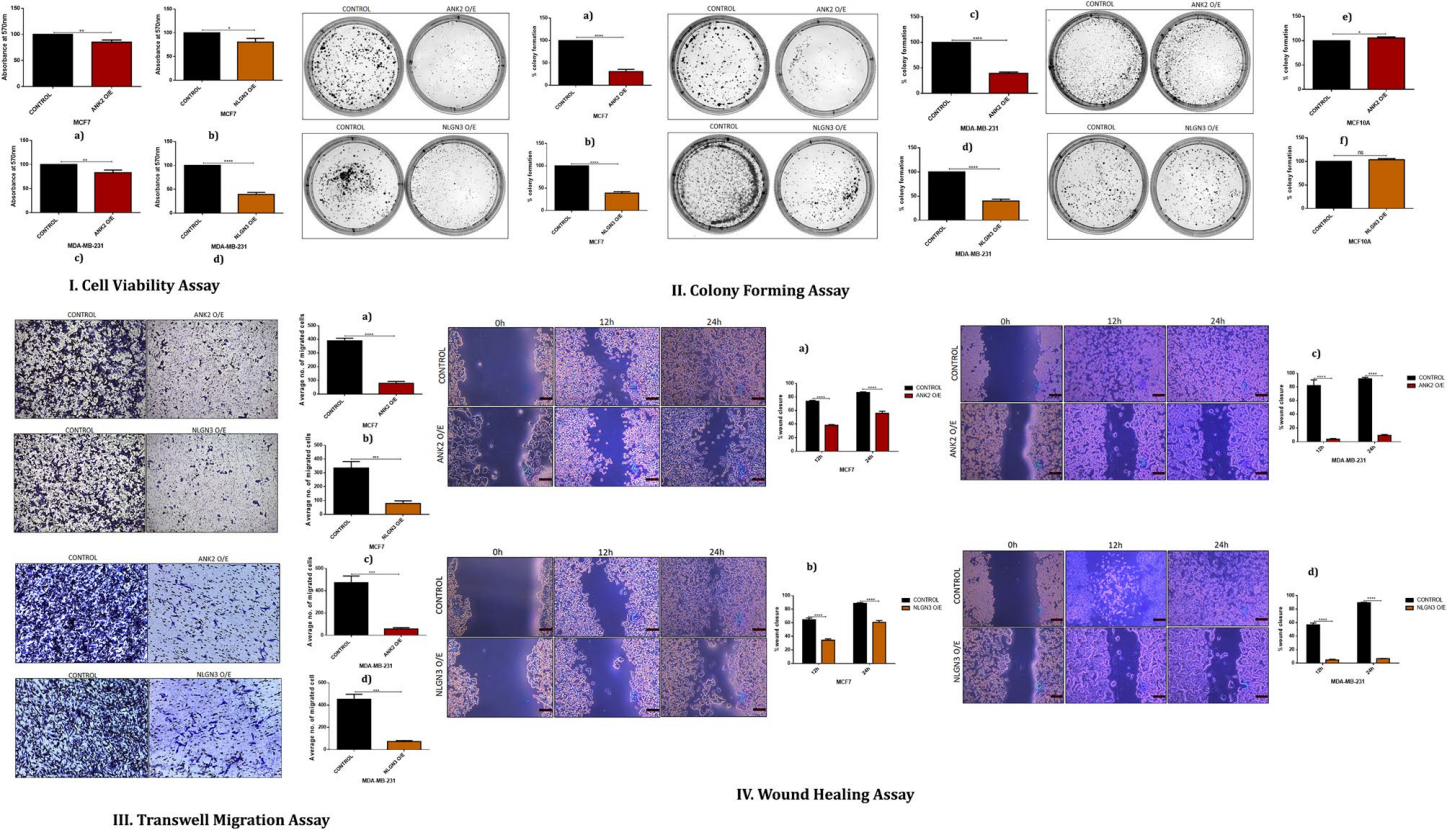
Overexpression of NLGN3 and ANK2 restricts breast cancer proliferation and migration. I. Cell proliferation quantified by MTT assay in control and NLGN3, ANK2 overexpressed MCF7 and MDA-MB-231 cells post 48hr. II. Overexpression of NLGN3 and ANK2 restricts breast cancer proliferation and migration. Colony forming assay showing number of colonies per plate along with graphical representation. III. Migration assay showing the number of migrated cells through trans-well chamber 48h after stimulation with 5% FBS. IV. Wound-healing showing 0 hr, 12 hr, and 24 hr post wounding and its graphical representation showing percentage wound closure in ctrl, ANK2O/E and NLGN3O/E-treated MCF7 and MDA-MB-231 cells. Data are represented as mean ± SD. 2 way ANOVA was used for the statistical analyses, n = 3 (****P < 0.0001,***P < 0.001, **P < 0.01, *P < 0.05).

To elucidate the involvement of ANK2 and NLGN3 in migratory and proliferative capacity of MCF7 and MDA-MB-231 cells, we performed transwell migration and cell proliferation assay and results showed ANK2 and NLGN3 overexpression decreased migration and proliferation of these cells. Next, colonogenic assay was performed to assess the impact of ANK2 and NLGN3 on colony formation, and the result showed small and a fewer numbers of colonies in both MCF7 and MDA-MB-231 cells overexpressing ANK2 and NLGN3 compared to control cells. Further, we investigated the role of ANK2 and NLGN3 in wound healing and found that ANK2 and NLGN3 overexpression impeded the migration of MCF7 and MDA-MB-231 cells (Fig. 6). Collectively, the in vitro findings suggest the role of ANK2 and NLGN3 in breast tumorigenesis suggesting it may be a potential drug target for breast cancer therapeutics.

### Proposed mechanism for the role of nesprin1 (SYNE1) protein in breast invasive carcinoma

**Figure 7:**
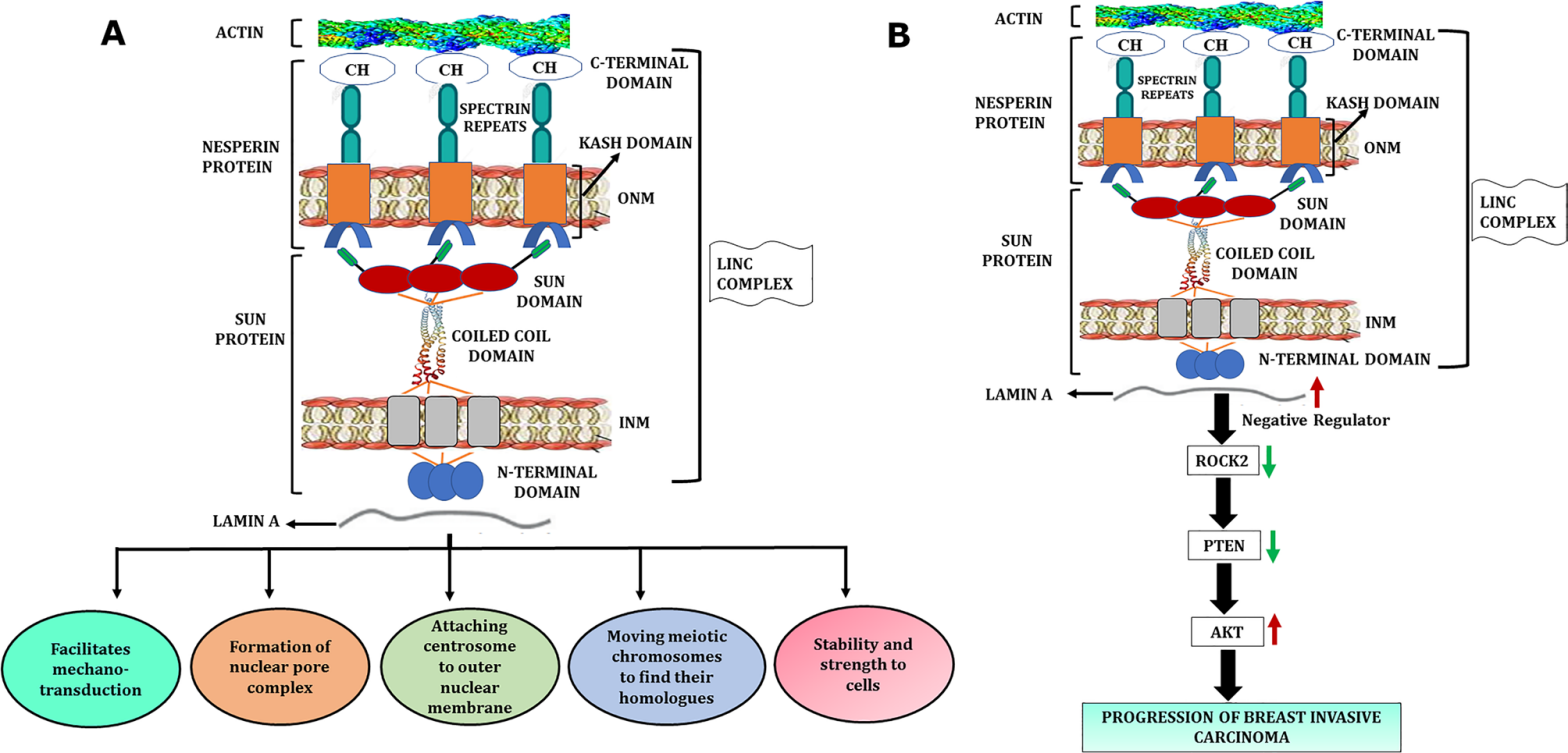
a) The functions of LINC complex in normal cells. b) The proposed role of nesprin 1 (SYNE1) in progression of breast invasive carcinoma.

The pathway describing the role of SYNE1 (Spectrin Repeat Containing Nuclear Envelope Protein 1) in the progression of breast invasive carcinoma has been proposed with the help of our bioinformatics analysis and detailed literature survey. SYNE is a protein that is found in the outer nuclear membrane which is comprised of N-terminal actin binding domain (ABD), a rod domain with variable spectrin repeats (SRs), and a KASH domain (KASH peptide and ONM (outer nuclear membrane) transmembrane domain) at C-terminal. At the ABD domain, SYNE 1/2 binds with actin and at the KASH peptide, it binds with SUN domain of SUN1/2 protein, forming a LINC (Linker of Nucleoskeleton and Cytoskeleton) complex. The N-terminal domain of SUN protein binds to nuclear lamina (LaminA). The LINC complex therefore links actin present in cytoplasm to nuclear lamina underlying the INM (inner nuclear membrane) and performs different cellular functions as (A) facilitates mechano-transduction, (B) formation of nuclear pore complex, (C) attaching centrosome to ONM D) provides stability and strength to cells (Fig. 7A) ^48,49^.

In the case of BRCA, there is a significant downregulation of SYNE1 protein as revealed by our analysis. This indicates that SYNE1 is less available to bind with SUN protein resulting in downregulation of LINC complex. Once the LINC complex is downregulated, the LaminA becomes free and its expression is upregulated. The LaminA has been reported to influence RhoA and its effector activity and acts as a negative regulator of ROCK2 protein ^50,51^. This suggests that as the LaminA is upregulated, the expression of ROCK2 gets down-regulated due to a negative feedback mechanism. The ROCK phosphorylates and activates PTEN which is a tumor suppressor gene ^52–54^. Therefore, when ROCK2 gets downregulated, PTEN is not able to get phosphorylated and remains inactive. PTEN is a phosphatase protein that dephosphorylates phosphatidylinositol-3,4,5-triphosphate (PIP3), which is the product of PI3K into phosphatidylinositol 4,5-bisphosphate (PIP2). The loss of PTEN function leads to the accumulation of PIP3 and enhanced activation of its subsequent downstream effectors as PDK1, AKT/PKB and CDK-cyclin. This eventually results in cell cycle progression, proliferation, cell survival and migration (Fig. 7B).

## Discussion

Understanding the molecular subtypes of breast cancer is important for predicting prognosis, guiding treatment decisions, and developing targeted therapies to improve outcomes for patients with breast cancer. Identification of dysregulated genes in different molecular subtypes can provide valuable insights into the underlying biological mechanisms driving the disease. It can also help in the development of personalized treatment approaches tailored to the molecular characteristics of the tumor.

Among the three molecular classes, TNBC is the most aggressive one. The five-year survival rate is lower for TNBC than other subtypes, perhaps due to lack of molecularly targeted therapies. Therefore, efforts are being done to develop new diagnostic and prognostic approaches. For example a ten gene expression signature has been reported to be associated with TNBC, in particular for Mexican patients ^55^. In TNBC different dysregulated non-coding RNAs were analyzed ^56^ and molecular pathways were identified ^57^. The overall and disease-free survival was related to certain gene expressions in TNBC ^58,59^.

The genomics differences between African and Caucasian American women were studied and 20 genes were identified that segregated two classes ^60^. ITGA11 and Jab1 were identified as biomarker for breast cancer ^61^. The mutations in important loci as BRCA1, BRCA2, PTEN, ATM, TP53, CHEK2, PPM1D, CDH1, MLH1, MRE11, MSH2, MSH6, MUTYH, NBN, PMS1, PMS2, BRIP1, RAD50, RAD51C, STK11 and BARD1 were related with risk of breast cancer ^62^. The relation between somatic mutations and prognosis was determined and mutations in PIK3R1 and DDR1 were found to be associated with poor outcomes in HR+ breast cancer ^63^.

This is the first study to identify subnetworks of genes in three molecular classes (ER/PR+/HER-2−, ER/PR-/HER-2+, and TNBC) in different clinical stages based on genetic dysregulation and mutations.

This study reports five genes viz. neuroligin3 (NLGN3), mastermind-like transcriptional coactivator 2 (MAML2), titin (TTN), ankyrin2 (ANK2) and spectrin repeat containing nuclear envelop protein 1 (SYNE1) to be significantly associated with distinct molecular classes of BRCA (ER/PR+/HER-2−, ER/PR-/HER-2+ and TNBC). The genes are involved in important processes such as chemotaxis and axon guidance, notch binding, cell adhesion molecule binding etc. They are central genes in the protein–protein-interaction (PPI) network indicating they can have important regulatory roles. The identified genes were found to be involved in different biological pathways such as TR/RXR activation pathway, Notch signaling pathway, regulation of EMT, Netrin signaling, RhoA signaling, axonal guidance signaling pathways, and galactose degradation I pathway. These pathways have important roles in cellular processes such as proliferation, differentiation, migration, survival and predispose oncogenesis.

In the early stage, pathways such as interferon signaling, tryptophan degradation III, granulocyte adhesion & diapedesis, and catecholamine biosynthesis were found in ER/PR+/HER-2− (p-value < 0.010). Whereas, pathways such as RAR activation, adipogenesis, the role of JAK1, JAK2, and TYK2 in interferon signaling, TGF-β signaling and STAT3 signaling (p-value < 0.014) were found enriched in ER/PR-/HER-2+. In the case of TNBC pathways such as IL-1/IL-8 signaling, TNFR1/TNFR2 signaling, NER, TWEAK signaling, and relaxin signaling (p-value < 0.005) were found to be enriched.

In late stages, calcium signaling, cholesterol biosynthesis, FXR/RXR activation, and AMPK signaling pathways were (p-value < 0.010) found to be associated with ER/PR/+/HER-2−. Whereas E1F2 signaling, mTOR signaling, renin-angiotensin, SAPK/JNK signaling and IL-10 signaling pathways (p-value < 0.014) were found enriched in TNBC.

One of the recent studies showed that genetic alterations in SYNE1 are found in 10% of gynecologic malignancies and 5% of epithelial ovarian cancers ^64^. Another study demonstrated that NLGN3 endorses glioma progression by upregulating LYN and ADAM10 activity, which in turn promotes NLGN3 cleavage to form a positive feedback loop ^65^. In a study by Cho et al.^66^ ANK2 was hypermethylated in canine mammary tumor and was also highlighted as potential tissue biomarker. Additionally, ANK2 showed significant hypermethylation in the plasma cfDNA of canine mammary tumors, indicating it could be a possible liquid biopsy biomarker as well.

Our findings were further validated using qRT-PCR, western blotting and immunohistochemistry which show decreased expression of NLGN3, MAML2, ANK2, and SYNE1 in breast cancer cell lines as MCF7 and MDA-MB-231 as compared to control cell line MCF10A. The tumor suppressor role of the selected genes ANK2 and NLGN3 was elucidated by cell viability assay, transwell migration, colony-forming and wound healing assay in MCF7, MDA-MB-231 and MCF10A cell lines. ANK2 and NLGN3 overexpression showed a reduction in migration and proliferation of MCF7 and MDA-MB-231 cells.

The present study revealed that the genes viz. NLGN3, MAML2, TTN, SYNE1, and ANK2 are significantly correlated with BRCA. They interact with numerous other genes associated with cell proliferation, survival, migration, and metastasis. The genes initiate and stimulate the proliferation of cancer cells by impeding the functions of downstream genes and disrupting normal cellular processes.

The results suggest that candidate genes may serve as useful biomarkers for different molecular classes of BRCA. The subnetworks containing gene components can be targeted for developing novel therapeutics for BRCA.

## Material and methods

**Figure 8:**
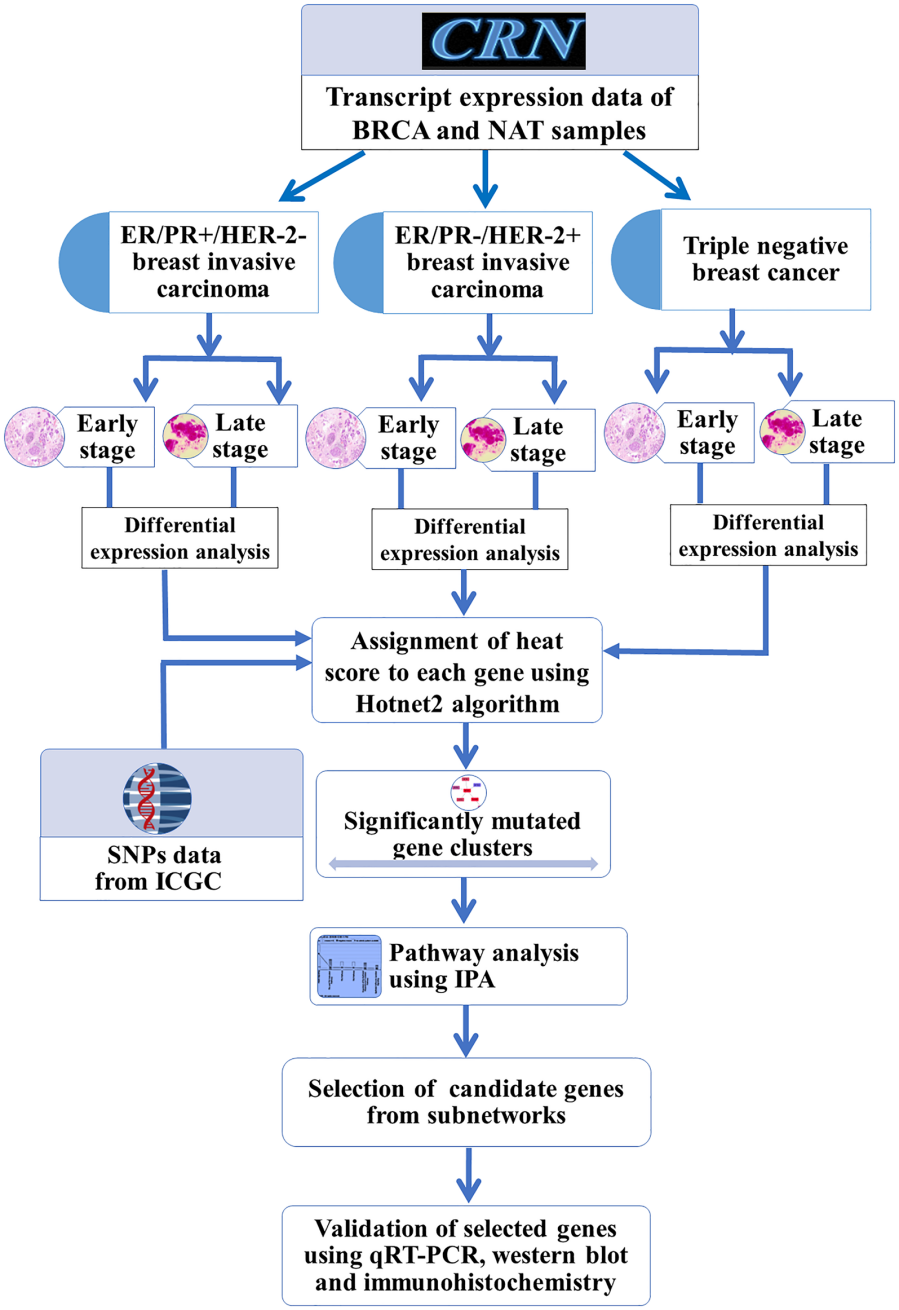
The flowchart of methodology. The Cancer RNA-seq Nexus (CRN) and International Cancer Genome Consortium (ICGC) were used as data sources as indicated in the figure. The differential expression analysis was performed on BRCA (breast invasive carcinoma) and NAT (Normal Adjacent Tissue) samples in three major molecular classes of BRCA in early and late stage. The genes were clustered into subnetworks based on their frequency of mutation. The pathway and functional enrichment analysis were performed on selected clusters of genes and selected genes were validated using qRT-PCR, western blot and immunohistochemistry. IPA=Ingenuity Pathway Analysis. qRT-PCR=Quantitative Real-Time Polymerase Chain Reaction. SNP=Single Nucleotide Polymorphism.

The different molecular classes of BRCA have been analyzed for genes that are differentially expressed in early and late stages, dysregulated biological pathways, and cancer progression using integrated computational analysis. The flowchart of the methodology is shown in Fig. 8.

### Data collection

The transcript expression data for BRCA tissue and normal adjacent tissue (NAT) was obtained from cancer RNA-seq nexus (CRN) (Jian et al. ^67^). The data contained transcripts expression values (total 71,361 (coding and non-coding)) in TPM (transcript-per-million base pairs) of 1097 tumor and 114 NAT samples. The non-coding (6287) transcripts were removed and the data was divided into three molecular classes (a) ER/PR+/HER-2− [tumor samples (362) vs NAT (37)], (b) ER/PR−/HER-2+ [tumor (36) vs NAT (04)] and (c) Triple negative breast cancer (TNBC) [tumor (113) vs NAT (10)]. Under each molecular class, data was further divided into clinical stages (stage I–IV as per standard TCGA classification). The number of tumor and NAT samples in each class is shown in Table 12. However, due to the small sample size in each stage, we classified data into two groups i.e. early (I, II) and late (III, IV) stage for the purpose of analysis. The transcripts with significant p-value (< 0.05) were kept for further analysis. The mean expression of transcripts that belong to a gene was considered as the expression for that gene.

**Table 12:** Summary of the number of samples obtained for BRCA (breast invasive carcinoma) and NAT (normal adjacent tissue) in each stage of ER/PR+/HER-2−, ER/PR−/HER-2+ and TNBC (triple negative breast cancer).

	ER/PR+/HER-2−		ER/PR−/HER-2+		TNBC	
	Stages	No. of samples (patients/NAT)	Stages	No. of samples (patients/NAT)	Stages	No. of samples (patients/NAT)
	I	39/7	I	01/00	I	09/01
	IA	32/1	IA	01/00	IA	10/01
	IB	01/00	IB	00/00	IB	00/00
	II	01/00	II	00/00	II	01/00
	IIA	111/11	IIA	11/01	IIA	51/03
	IIB	91/8	IIB	12/03	IIB	21/03
Early stage	Total samples	275/27	Total samples	25/4	Total samples	92/8
	IIIA	53/6	IIIA	05/00	IIIA	13/01
	IIIB	08/01	IIIB	00/00	IIIB	03/00
	IIIC	22/02	IIIC	05/00	IIIC	03/01
	IV	03/01	IV	01/00	IV	02/00
Late stage	Total samples	86/10	Total samples	11/00	Total samples	21/02

### Differential expression study

The fold change was calculated using TPM values in tumor and NAT. The genes with a log_2_ FC greater than or less than 1 and p-value < 0.05 were considered up or downregulated respectively ^68^. The differential expression pattern was studied in the early and late stages in each of the three class. Further, due to non-availability of NAT sample in late ER/PR−/HER-2+ class, it was removed from the study. Finally, five classes (1) Early stage ER/PR+ /HER-2−. (2) late stage ER/PR+/HER-2−. (3) Early stage ER/PR−/HER-2+. (4) Early stage TNBC and (5) Late stage TNBC were analyzed in detail. The biological pathways in each class were also analyzed to check similarities and differences in terms of affected pathways in different classes.

### Clustering of genes into subnetworks

The genes were clustered based on differential expression, protein–protein interaction (PPI) and mutational frequency using Hotnet2 algorithm. The hotnet2 is an algorithm to find out mutated clusters and overcomes limitations with existing algorithms as MEMo, HotNet etc. These clusters can have co-occurring mutations across cancer samples and their component genes may work together with upstream/downstream genes to drive biological pathway(s). The purpose was to uncover modules, pathways, and processes which are getting affected in different classes of BRCA. The mutation data for BRCA was collected from ICGC ^66^ and subnetworks were created using Hotnet2 algorithm (supplementary data 1).

### Pathway analysis

Pathway analysis was performed using IPA (Ingenuity-Pathway-Analysis), RRID: SCR_008653, using p-value cutoff of 0.05 to uncover functional role of various modules (subnetworks).

### Quantitative-real-time polymerase-chain-reaction (qRT-PCR)

Breast cancer cell lines MCF7, T47D and MDA-MB-231 were purchased from National Centre for Cell Sciences (NCCS), Pune, India and MCF10A was a kind gift from Dr. Annapoorni Rangarajan (IISC, Bangalore, India). The total RNA was isolated from MCF7, T47D, MDA MB-231 and MCF10A cells using Tri-reagent (Sigma-Aldrich). Reverse transcription PCR and qRT-PCR was performed using primers of NLGN3, MAML2, SYNE1 and ANK2. The detailed methodology and primer sequences are given in supplementary data 2. GAPDH was taken as an internal control and ΔΔCT values were calculated. The statistical analysis was performed using GraphPad Prism (RRID: SCR_002798), which includes unpaired student’s t-test for estimation of statistical significance.

### Western blot analysis

The whole cell lysate of MCF7, MDA-MB-231 and MCF10A were prepared using RIPA buffer. The samples were run in 10% SDS-PAGE gel, transferred on PVDF membrane (Millipore) and blocked with 5% (w/v) non-fat milk (Sigma). Blots were then incubated with primary antibody (ANK2, SYNE1, NLGN3) overnight at 4 °C with gentle shaking. The membrane was then washed with 1× TBS-T and incubated with anti-rabbit or anti-mouse horseradish peroxidase-conjugated secondary antibody (1:5000 dilution) for 1 h. After washing, the blots were developed using Western Blot Chemiluminescence HRP Substrate (Takara Bio Inc.) in Chemidoc XRS+ molecular 228 imager (Bio-Rad, Hercules, CA, USA). The images were quantified using Image J software. The detailed methodology is given in supplementary data 3.

### Immunohistochemistry

The tissue microarray slides (TMA) were purchased from US Biomax for each of the genes (NLGN3, MAML2, SYNE1 and ANK2, RRID: AB_1857707 and RRID: AB_2667965). Each slide contained a hundred samples of invasive ductal carcinoma and nine samples of normal adjacent tissue. The samples belonged to different molecular classes, clinical stages, ages and grades. Corresponding to our analysis, we have divided the samples into four categories i.e. early stage ER/PR+/HER-2−, late stage ER/PR+/HER-2−, early stage TNBC and late stage TNBC. The number of samples in early stage ER/PR+/HER-2−, late stage ER/PR+/HER-2−, early stage TNBC and late stage TNBC were 23, 7, 13, and 3 respectively. There are 57 samples of grade 2 and 42 samples are of grade 3. The age-wise distribution was as follows: 23 (< = 40 years), 44 (41–50 years), 22 (51–60 years), 11 (> 60 years).

Four slides were stained by anti-MAML2, anti-ANK2, anti-SYNE1 and anti-NLGN3 antibodies separately at 1:50 dilution and were further processed using the ABC system (Vector Laboratories, Bulingame, CA, USA) as described previously ^67^. The images were captured under Leica microscope using LAS EZ software version 2.1.0 at 40× magnification. The tissue spots were scored by pathologist in a blinded experiment. The score for the intensity of staining and percentage of positive cells were given as per the Allred scoring system. The staining intensity was scored as: 0 for negative, 1 for weak, 2 for intermediate and 3 for strong. The percentage positive cells were scored between 0 and 100%: 0 for no cells, 1 for < 1% cells, 2 for 1–10%, 3 for 11–33%, 4 for 34–66% and 5 for 67–100%. Quick score for each sample was calculated by multiplying the intensity score by score for percentage positive cells. The statistical analysis was done with quick score and graph was plotted.

### Cell viability assay

Cell viability assay was performed to check the proliferation rate of MCF7 and MDA-MB-231 control upon NLGN3 and ANK2 overexpression. Control and overexpressed MCF7 and MDA-MB-231 cells were seeded at a density of 3 × 10^3^ cells per well in 96-well plates. Cells were incubated for 48 h. After the stipulated time, 10 μl of 3-(4,5-dimethylthiazol-2-yl)-2,5-diphenyltetrazolium bromide (MTT) at a concentration of 5 mg/ml were added into each well and incubated for 4 h. After incubation, the media was removed and 100 μl of DMSO was added to dissolve the purple formazan crystals. The absorbance was taken at a wavelength of 570 nm in a Varioskan™ flash multimode reader (Thermo Scientific). Three independent sets of experiments were performed. The percent viability was calculated by the formula- %viability = A/A_0_ where A_0_ and A are the absorbances of the vehicle control and NLGN3/ANK2 overexpresses cells respectively.

### Colony forming assay

For colony forming assay, briefly after transfecting of MCF7 and MDA-MB-231 cells with NLGN3 and ANK2, 0.6 × 10^3^ control, NLGN3, and ANK2 overexpressed cells were seeded in triplicates in 60 mm-plates. The plates were kept at 37 °C, 5%CO2 chamber for 1 week to allow the growth of colonies (50 cells per colony). Then the cells were washed with 1× PBS, fixed with 10%(v/v) formalin, and then stained with 0.01%(w/v) crystal violet solution. Excess stain was removed by washing twice with 1XPBS. The plate was air-dried followed by imaging in Gel Doc™ XR + Imager (Bio-Rad). The stained cells were dissolved in 10% (v/v) acetic acid and the absorbance was quantified at 570 nm using Varioskan™ Flash Multimode Reader (Thermo Scientific). Colony formation rate was calculated by the formula—Colony forming rate = 100% × (experimental absorbance value/control absorbance value).

### Transwell migration assay

Transwell migration assay was performed following manufacturer’s protocol (BD Falcon, USA).) 48 h post-transfection, control, NLGN3 and ANK2 overexpressed MCF7 and MDA-MB-231 cells were seeded at a density of 0.5 × 10^3^ cells in upper chamber of 24 well trans-well system in 500 μl of DMEM media. Medium supplemented with 5% serum was used as a chemoattractant in the lower chamber. After 24 h the cells on both sides of the membrane were fixed with 10% formalin and stained with 0.01% crystal violet stain. The membrane was then washed with PBS and the cells attracted towards the serum were visualized under a light microscope and pictured (10×) under different field views. The number of migrated cells in control and overexpressed cells in 10 different fields were calculated using ImageJ software and the average value was represented in the graph.

### Wound healing assay

1 × 10^4^ MCF7 and MDA-MB-231 control, NLGN3 and ANK2 overexpressed cells were plated and grown up to 90% confluence in a 12-well plate (Falcon Becton Dickinson). Cells were then scratched with a sterile 200 μl pipette tip in each well. The cells were washed twice with 1× PBS and the image was captured such as cells at stage 1 that is 0 h. Images of the cells undergoing migration were then taken at different time points at a magnification of 10×. Quantitation of migrated cells was done by calculating the decrease in the area at all the observed time points with the help of ImageJ software.

## Conclusion

Our integrated approach has enabled the identification of candidate genes in ER/PR+/HER-2−, ER/PR-/HER-2+, and TNBC in the early and late stages of BRCA. We found some interesting patterns e.g. genes which were upregulated in one molecular class, were downregulated in another class, and vice-versa. Additionally, some genes were found to be class specific. This difference might be due to differences in expression patterns of hormone receptors among the molecular classes. Significantly mutated subnetworks containing dysregulated, mutated and driver genes were also identified in different molecular classes of BRCA which gave insight into their biological functions. We believe that the dysregulated genes can serve as biomarkers for early detection of the class and stage of BRCA. Our approach seeks to maximize the use of datasets and techniques to understand the role of coding genes in disease pathogenesis, prognosis, and the development of diagnostic tools and therapeutics. We hope that identifying genes and their subnetworks will contribute to drug design and discovery.

### Supplementary Information

Supplementary Information 1.

## Data Availability

The datasets used and/or analysed during the current study available from the corresponding author on reasonable request.
