# Supplementary material for: Dysregulated gene subnetworks in breast invasive carcinoma reveal novel tumor suppressor genes
Source: Sci Rep. 2024 Jul 8;14:15691. doi: 10.1038/s41598-024-59953-0 (PMC11231308; doi:10.1038/s41598-024-59953-0)

Supplementary information includes tables S1-S4, figures S1-S7 and supplementary data which includes detailed methodological information

**Full length western blots (figure 4) illustrates the expression level of ANK2, SYNE1, and NLGN3 in in different breast cancer cell lines (MDA-MB-231 and MCF-7) and normal breast epithelial cell line (MCF10A).**


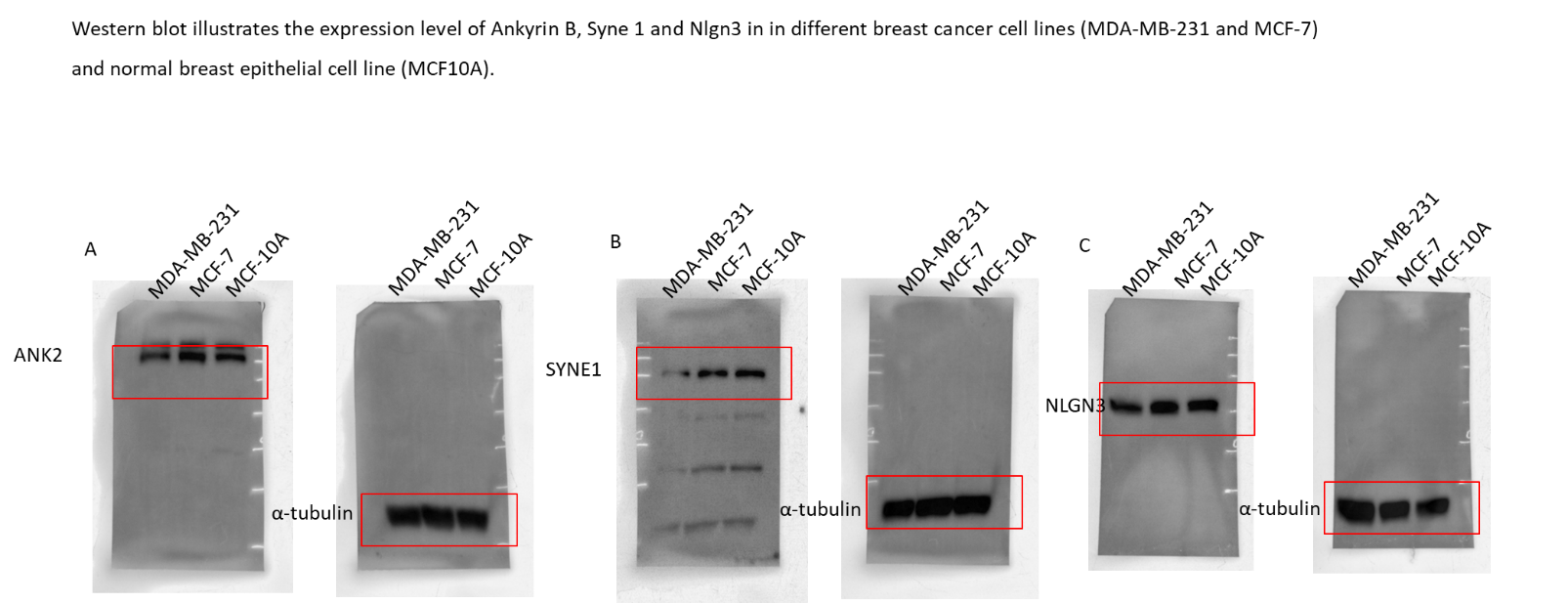

Supplement: Supplementary file 1 — Supplementary Information 1. [file 41598_2024_59953_MOESM1_ESM.zip › Supplementary information.docx]
