# Supplementary material for: Dysregulated gene subnetworks in breast invasive carcinoma reveal novel tumor suppressor genes
Source: Sci Rep. 2024 Jul 8;14:15691. doi: 10.1038/s41598-024-59953-0 (PMC11231308; doi:10.1038/s41598-024-59953-0)
Supplement: Supplementary file 1 — Supplementary Information 1. [file 41598_2024_59953_MOESM1_ESM.zip › Supplementary tables legends.docx]

**Supplementary table legends:**

Supplementary table S1a: List of differentially expressed genes in early stage ER/PR+/HER-2-.

Supplementary table S1b: List of differentially expressed genes in late stage ER/PR+/HER-2-.

Supplementary table S1c: List of differentially expressed genes in early stage_ER/PR-/HER-2+.

Supplementary table S1d: List of differentially expressed genes in early stage triple negative breast cancer.

Supplementary table S1e: List of differentially expressed genes in late stage triple negative breast cancer.

Supplementary table S2a: Overlap of dysregulated genes among different classes.

Supplementary table S2b: List of differentially expressed genes specific to class.

Supplementary table S2c: The inverse expression pattern of genes that were found among different classes.

Supplementary table S3a: Biological pathways in early stage ER/PR+/HER-2-.

Supplementary table S3b: Biological pathways in late stage ER/PR+/HER-2-.

Supplementary table S3c: Biological pathways in early stage ER/PR-/HER-2+.

Supplementary table S3d: Biological pathways in early stage TNBC.

Supplementary table S3e: Biological pathways in late stage TNBC.

Supplementary table S4a: Mutations present in components of cluster in early stage ER/PR+/HER-2-.

Supplementary table S4b: Mutations present in components of cluster in early stage ER/PR-/HER-2+.

Supplementary table S4c: Mutations present in components of cluster in early stage triple negative breast cancer.

Supplementary table 4d: Mutations present in components of cluster in late stage triple negative breast cancer.
