# Supplementary material for: Dysregulated gene subnetworks in breast invasive carcinoma reveal novel tumor suppressor genes
Source: Sci Rep. 2024 Jul 8;14:15691. doi: 10.1038/s41598-024-59953-0 (PMC11231308; doi:10.1038/s41598-024-59953-0)
Supplement: Supplementary file 1 — Supplementary Information 1. [file 41598_2024_59953_MOESM1_ESM.zip › Supplementary_fig.S1.pdf]

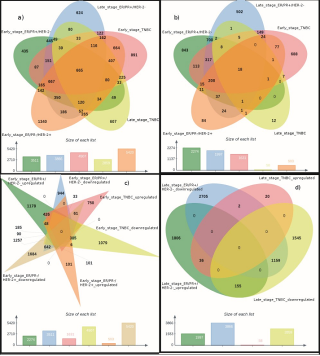


Supplementary figure 1. The dysregulated genes among different classes a) Stage specific downregulated genes among different classes. Total 665 genes were found to be downregulated in all categories i.e. Early stage ER/PR+/HER-2-; Late stage ER/PR+/HER-2-; Early stage ER/PR-/HER-2+; Early stage TNBC and Late stage TNBC. b) The overlap of upregulated genes among different classes. The 18 genes were found to be commonly upregulated in all five classes Early stage ER/PR+/HER-2-; Late stage ER/PR+/HER-2-; Early stage ER/PR-/HER-2+; Early stage TNBC and Late stage TNBC. Commonly dysregulated genes in early (c) and late stage (d) The inverse expression pattern was accorded for 38 genes which were downregulated in Early stage ER/PR+/HER-2- and upregulated in early stage TNBC. Similarly, genes showed inverse patterns among other classes as well. The detailed information is given in table 3 and supplementary table S2a, S2b and S2c.
