# Supplementary material for: Dysregulated gene subnetworks in breast invasive carcinoma reveal novel tumor suppressor genes
Source: Sci Rep. 2024 Jul 8;14:15691. doi: 10.1038/s41598-024-59953-0 (PMC11231308; doi:10.1038/s41598-024-59953-0)
Supplement: Supplementary file 1 — Supplementary Information 1. [file 41598_2024_59953_MOESM1_ESM.zip › Supplementary_fig.S2.pdf]

TR/RXR Activation pathway

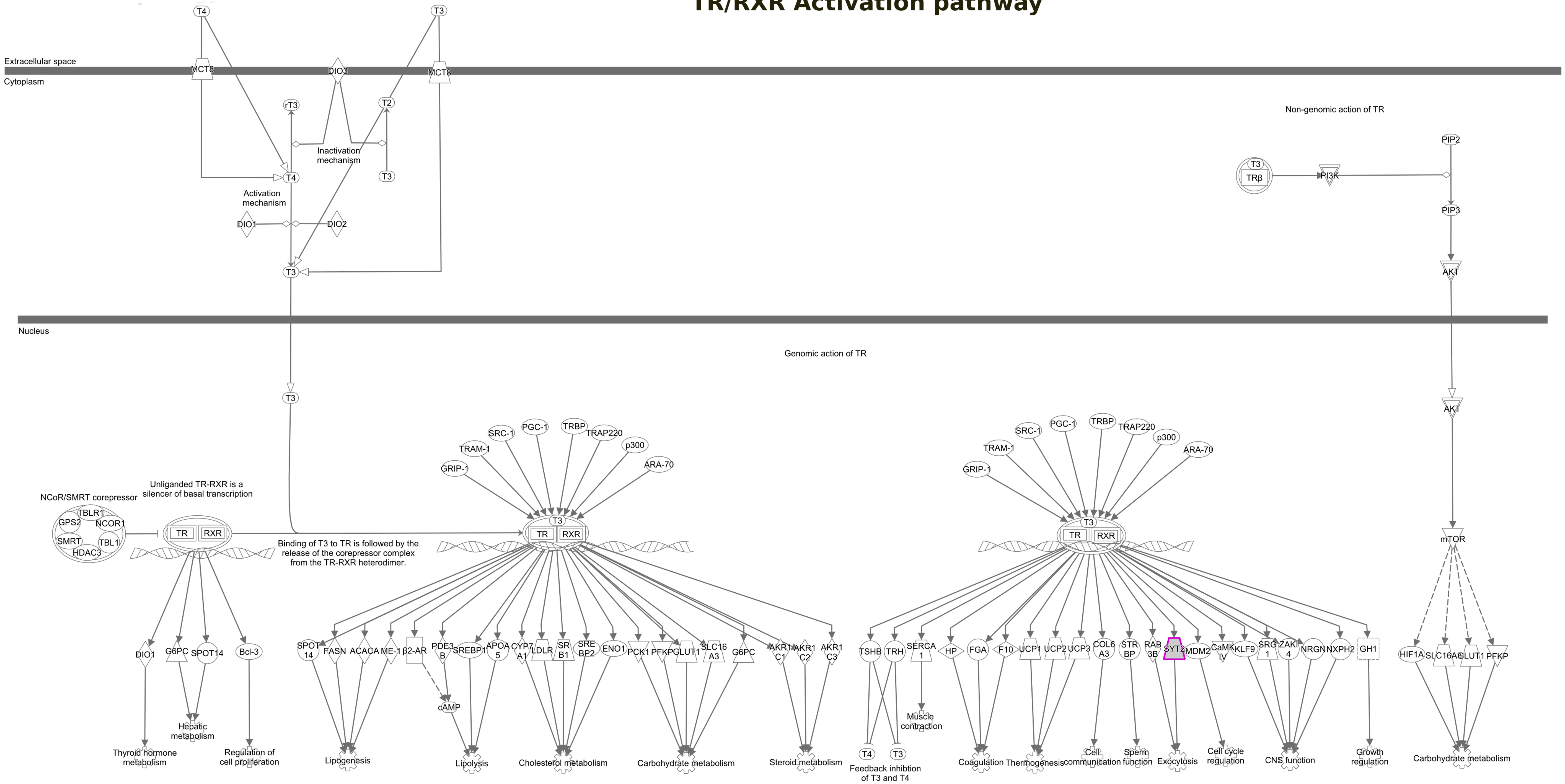

Supplementary Figure S2. Figure showing pathway diagram of TR/RXR activation pathway mediated by NLGN3, NRXN1, NLGN1 network and found to be involved in early stage ER/PR+/HER-2- class of breast invasive carcinoma with p-value= 3.43e-03.
