# Supplementary material for: Dysregulated gene subnetworks in breast invasive carcinoma reveal novel tumor suppressor genes
Source: Sci Rep. 2024 Jul 8;14:15691. doi: 10.1038/s41598-024-59953-0 (PMC11231308; doi:10.1038/s41598-024-59953-0)
Supplement: Supplementary file 1 — Supplementary Information 1. [file 41598_2024_59953_MOESM1_ESM.zip › Supplementary_fig.S3b.pdf]

TH1 and TH2 Activation pathway

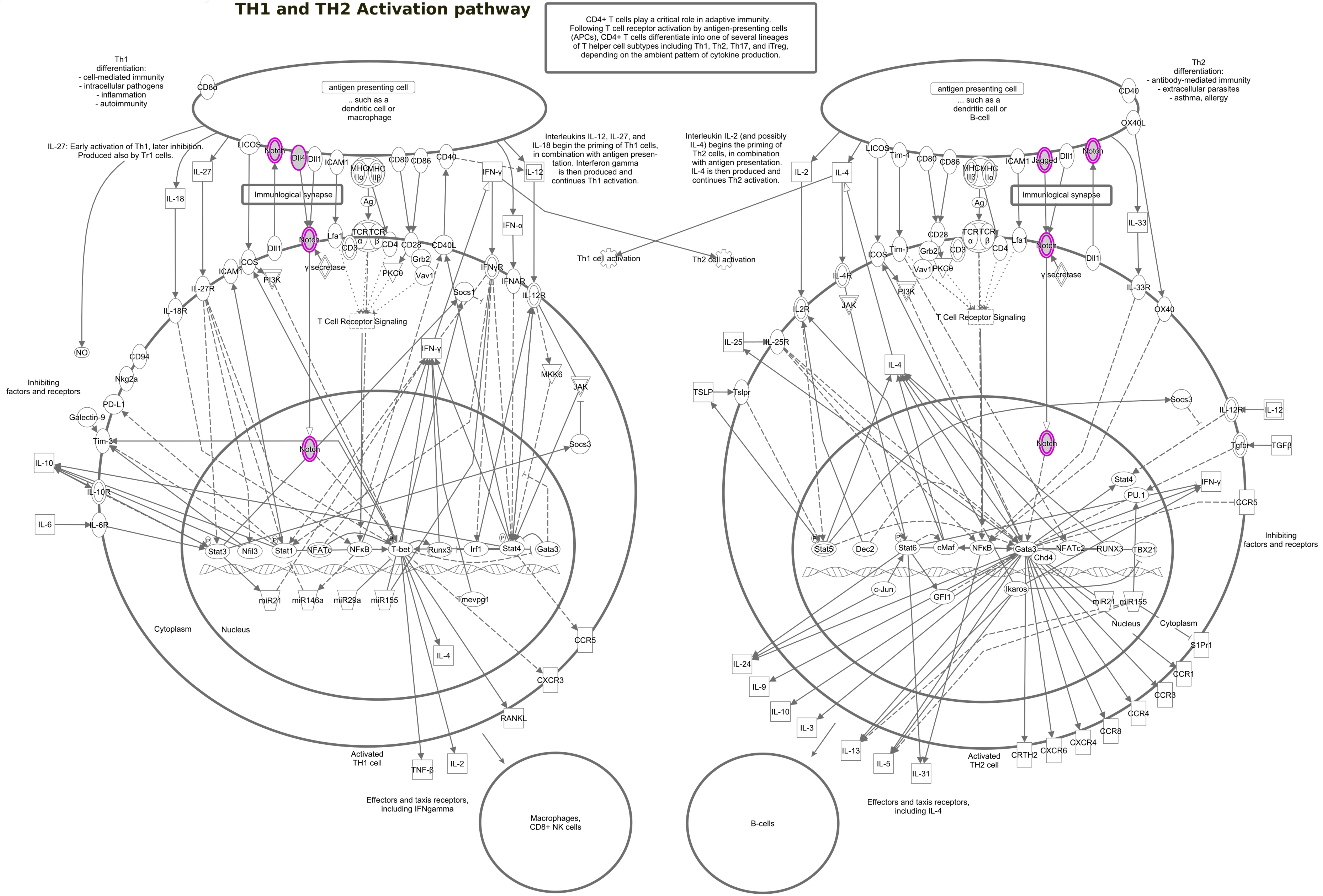

Supplementary Figure S3b. Figure showing pathway diagram of Th1 and Th2 activation pathway mediated by NOTCH genes and interactors and found to be involved in early stage ER/PR-/HER-2+ class of breast invasive carcinoma with p-value 1.00e-07.
