# Supplementary material for: Dysregulated gene subnetworks in breast invasive carcinoma reveal novel tumor suppressor genes
Source: Sci Rep. 2024 Jul 8;14:15691. doi: 10.1038/s41598-024-59953-0 (PMC11231308; doi:10.1038/s41598-024-59953-0)
Supplement: Supplementary file 1 — Supplementary Information 1. [file 41598_2024_59953_MOESM1_ESM.zip › Supplementary_fig.S3c.pdf]

# Regulation of the Epithelial Mesenchymal Transition pathway

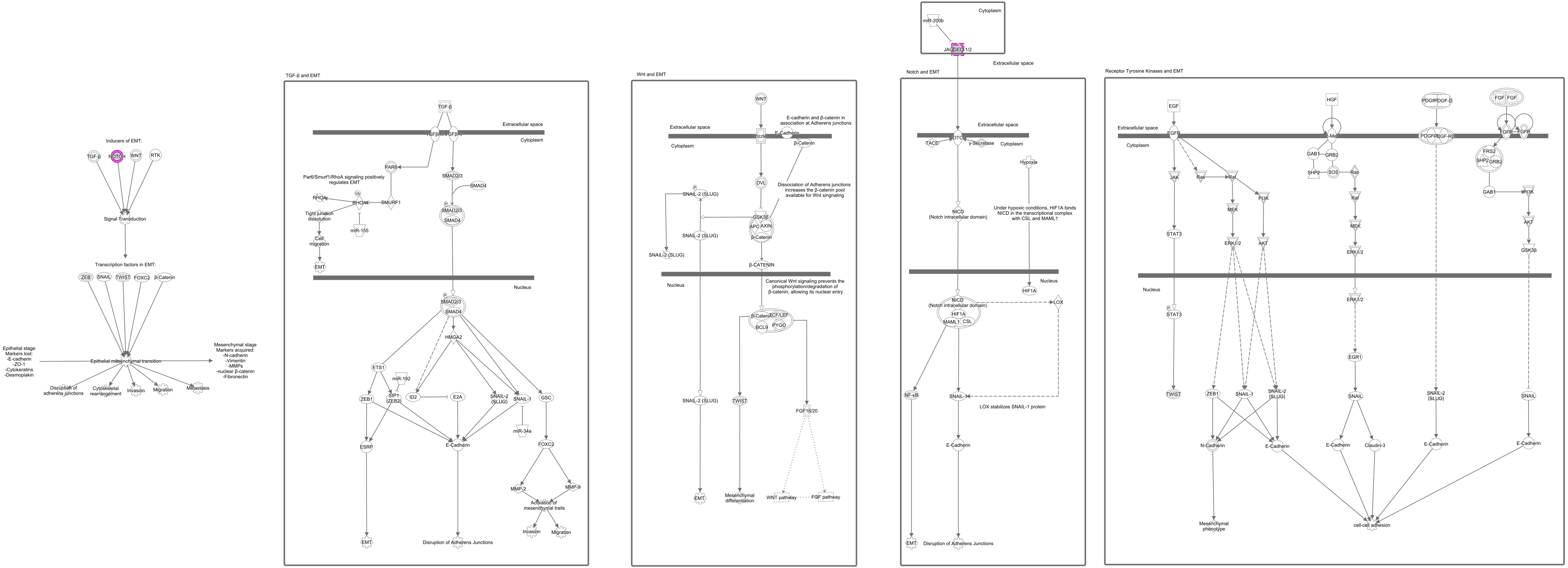


**Supplementary Figure S3c. Figure showing pathway diagram of Epithelial mesenchymal transition (EMT) pathway mediated by NOTCH genes and interactors and found to be involved in early stage ER/PR-/HER-2+ class of breast invasive carcinoma with p-value 1.82e-05.**
