# Supplementary material for: Dysregulated gene subnetworks in breast invasive carcinoma reveal novel tumor suppressor genes
Source: Sci Rep. 2024 Jul 8;14:15691. doi: 10.1038/s41598-024-59953-0 (PMC11231308; doi:10.1038/s41598-024-59953-0)
Supplement: Supplementary file 1 — Supplementary Information 1. [file 41598_2024_59953_MOESM1_ESM.zip › Supplementary_fig.S4a.pdf]

Netrin Signaling

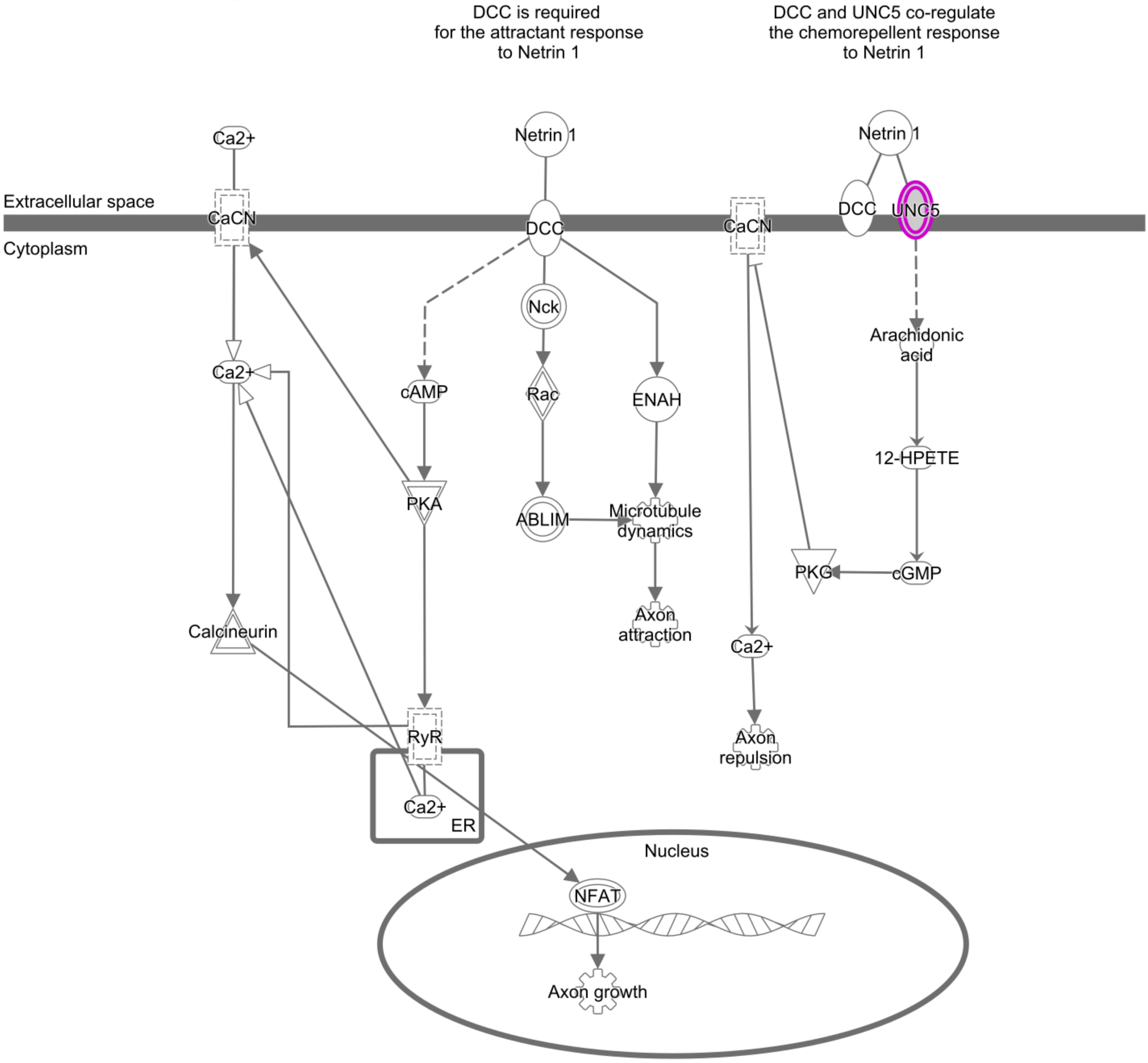

Supplementary Figure S4a. Figure showing pathway diagram of netrin signalling mediated by TTN and interactors and found to be involved in early stage TNBC with p-value 2.61e-02.
