# Supplementary material for: Dysregulated gene subnetworks in breast invasive carcinoma reveal novel tumor suppressor genes
Source: Sci Rep. 2024 Jul 8;14:15691. doi: 10.1038/s41598-024-59953-0 (PMC11231308; doi:10.1038/s41598-024-59953-0)
Supplement: Supplementary file 1 — Supplementary Information 1. [file 41598_2024_59953_MOESM1_ESM.zip › Supplementary_fig.S5b.pdf]

Colanic Acid Building Blocks Biosynthesis

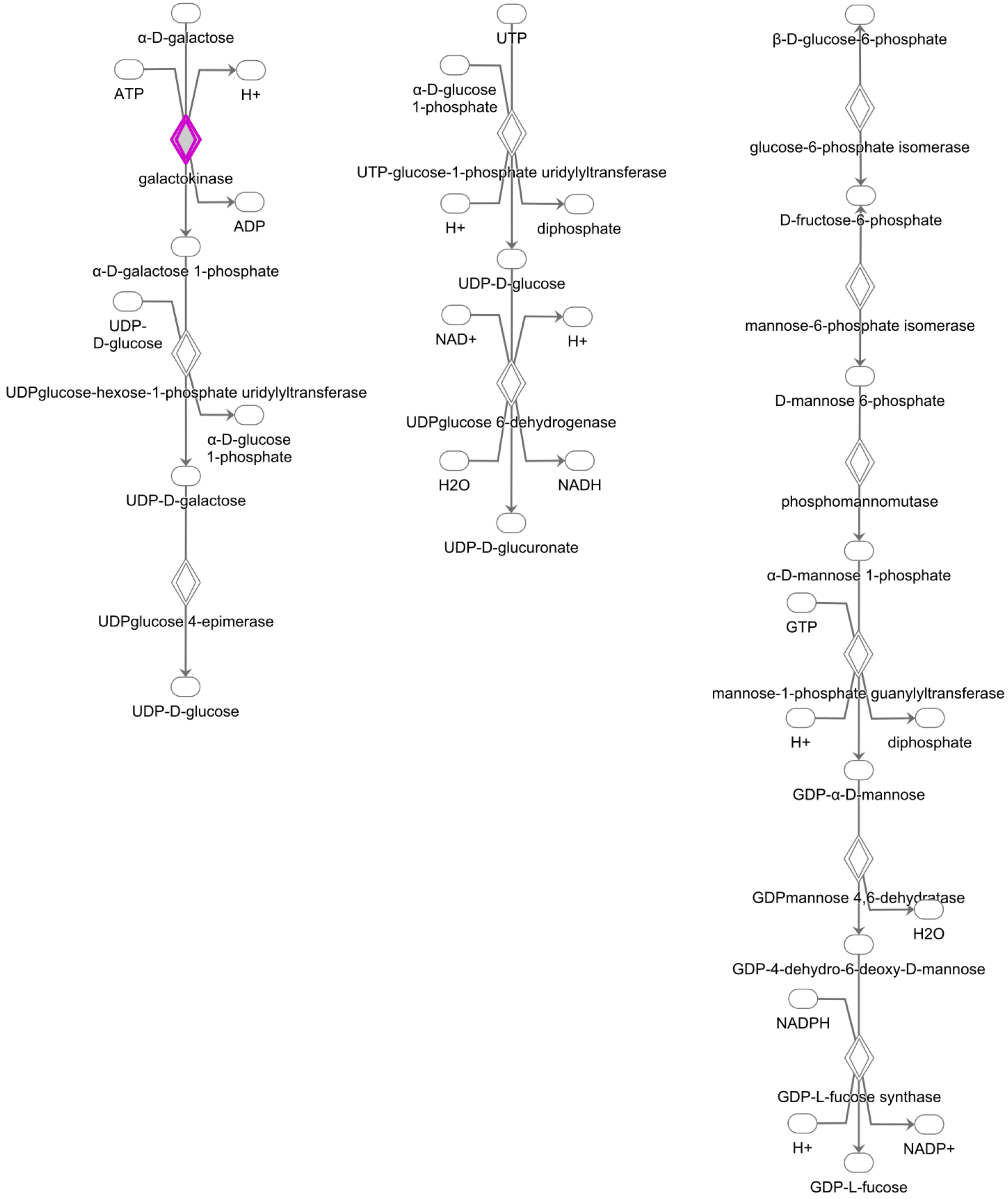

Supplementary Figure S5b. Figure showing pathway diagram of Colanic acid pathway mediated by DMD, ANK1, ANK2 and interactors and found to be involved in late stage TNBC with p-value 9.91e-03.
