# Supplementary material for: Dysregulated gene subnetworks in breast invasive carcinoma reveal novel tumor suppressor genes
Source: Sci Rep. 2024 Jul 8;14:15691. doi: 10.1038/s41598-024-59953-0 (PMC11231308; doi:10.1038/s41598-024-59953-0)
Supplement: Supplementary file 1 — Supplementary Information 1. [file 41598_2024_59953_MOESM1_ESM.zip › Supplementary_fig.S6.pdf]

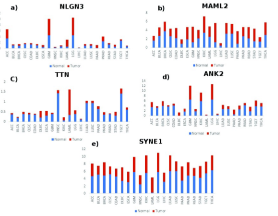

Supplementary Figure S6. Expression profile of selected genes in the tumor (red) and normal tissues (blue) across different cancers. The X-axis represents normal and tumor samples and Y-axis represents median expression (p-value ≤ 0.01). The median expression values are the log2(TPM + 1) values. ACC; Adrenocortical carcinoma, BLCA; Bladder Urothelial Carcinoma, BRCA; breast invasive carcinoma, CESC; cervical squamous cell carcinoma and endocervical adenocarcinoma, COAD; colon adenocarcinoma, DLBC; Lymphoid Neoplasm Diffuse Large B-cell Lymphoma, ESCA; esophageal carcinoma, GBM; Glioblastoma multiforme, HNSC; Head and neck squamous cell carcinoma, KIRC; Kidney renal clear cell carcinoma, LAML; Acute Myeloid Leukemia, LGG; Brain Lower Grade Glioma, LIHC; liver hepatocellular carcinoma, LUAD; lung adenocarcinoma, LUSC; lung squamous cell carcinoma, PAAD; Pancreatic adenocarcinoma; PRAD; Prostate adenocarcinoma, READ; Rectum adenocarcinoma, STAD; stomach adenocarcinoma, THCA; Thyroid carcinoma, TGCT; Testicular Germ Cell Tumors, TPM; Transcripts per Million.
