# Supplementary material for: Dysregulated gene subnetworks in breast invasive carcinoma reveal novel tumor suppressor genes
Source: Sci Rep. 2024 Jul 8;14:15691. doi: 10.1038/s41598-024-59953-0 (PMC11231308; doi:10.1038/s41598-024-59953-0)
Supplement: Supplementary file 1 — Supplementary Information 1. [file 41598_2024_59953_MOESM1_ESM.zip › Supplementary_fig.S7.pdf]

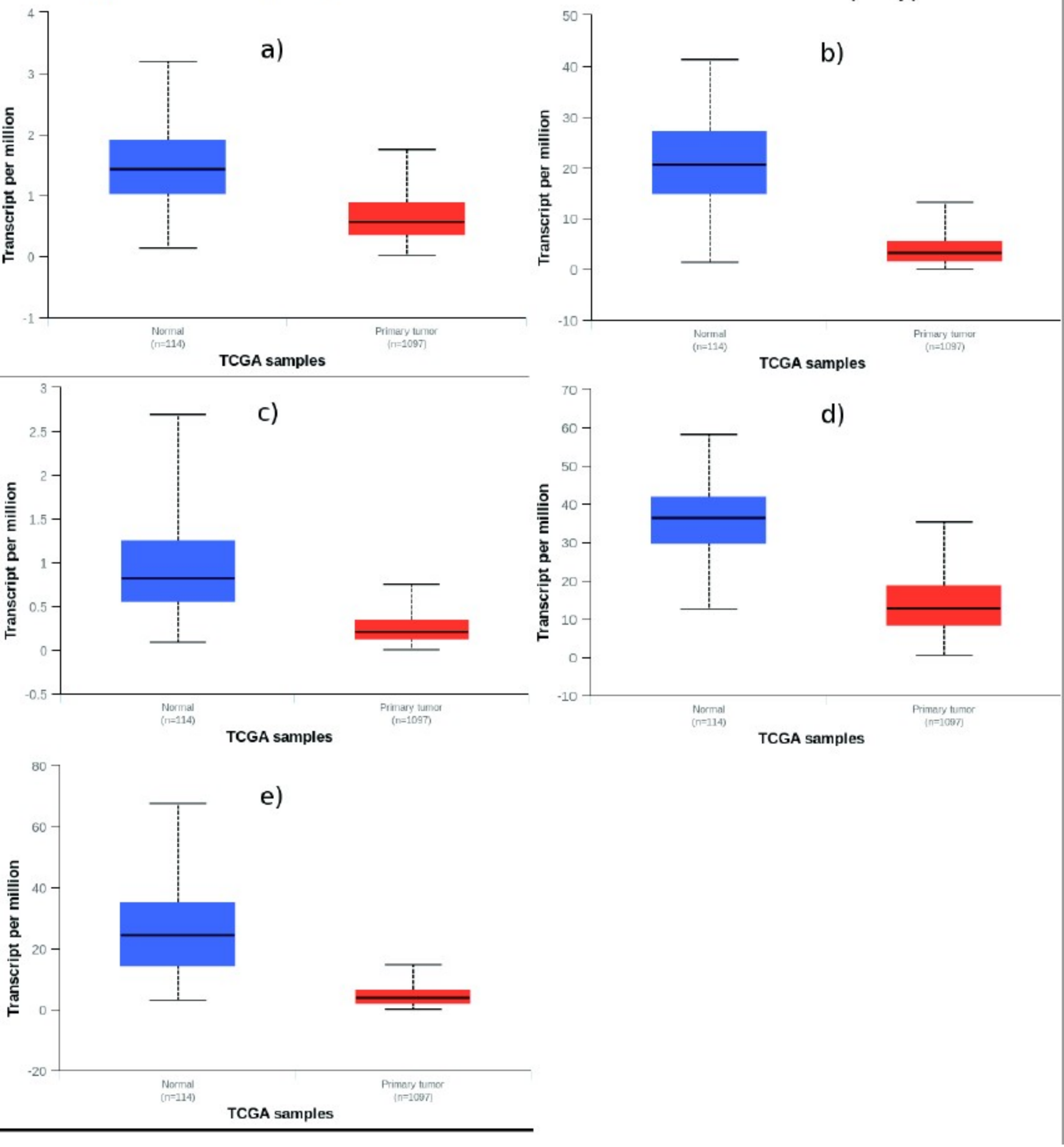

Supplementary figure S7. Figure showing differential expression of genes a) NLGN3, b) MAML2 c) TTN d) SYNE1 and e) ANK2 from UALCAN. The x-axis represents normal and tumor samples and y-axis represents transcript per million values. All genes are downregulated in tumor samples as compared to normal samples.
