# Supplementary material for: Dysregulated gene subnetworks in breast invasive carcinoma reveal novel tumor suppressor genes
Source: Sci Rep. 2024 Jul 8;14:15691. doi: 10.1038/s41598-024-59953-0 (PMC11231308; doi:10.1038/s41598-024-59953-0)
Supplement: Supplementary file 1 — Supplementary Information 1. [file 41598_2024_59953_MOESM1_ESM.zip › TMA-slides-Metadata_sheet.pdf]

BC081120d : Breast cancer with breast tissue array, including pathology grade, TNM, clinical stage and IHC marker (ER, PR and Her-2), 110 cases/110 cores, replaced by BC(

Microarray Panel	Breast carcinoma with breast tissue microarray, containing 99 cases of invasive ductal carcinoma, 1 intraductal carcinoma, 9 adjacent normal breast tissue, 1 cancer adjacent breast tissue, si case
Cores	110
Cases	110
Row number	11
Column number	10
Core Diameter (mm)	1
Thickness (μm)	5
Quantity Control	H&E and IHC confirmed
Tissue Array Type	FFPE
Species	Human
Applications	54
Notes	1. TMA slides were sectioned and stored at 4°C and may not be fresh cut, but still suitable for IHC. Please request fresh cut if experiment involves phospho-specific antibodies, RNA studies, A minimum of 3 slides per TMA must be purchased to cover the cost of trimming for fresh sectioning. 2. Most TMA slides were not coated with an extra layer of paraffin (tissue cores can be e the glass). **To prevent tissue detachment during antigen retrieval, unbaked slides must be baked for at least 30 to 120 minutes at 60°C**. before putting into xylene for de-paraffinizatio were sent out baked for 2 hours.<br>In the following specsheet,"*" means invalid core; "-" means no applicable or negative in IHC markers.

BC081120d H&E

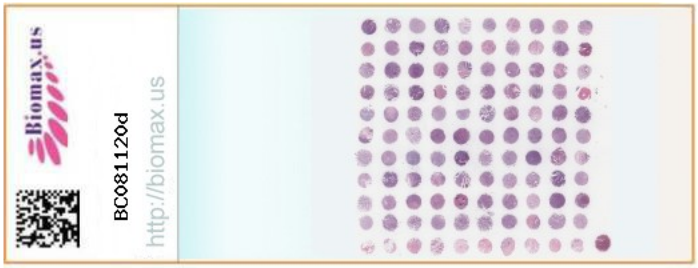

Mouseover and click individual cores to view high resolution images.

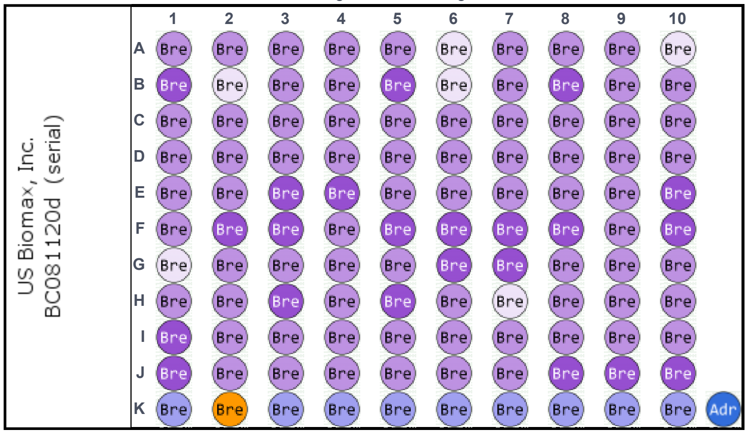

Legend: Bre - Breast

- AT, - Malignant tumor, - Malignant tumor (stage IA), - Malignant tumor (stage IIA), - Malignant tumor (stage IIB), - Malignant tumor (stage IIIA), - Malignant tumor (stage IIIB
, tissue IDs are available in exported Excel files.

Pos.	No.	Age	Sex	Organ/Anatomic Site	Pathology diagnosis	TNM	Grade	Stage	Type	ER	PR	HI
A1	1	43	F	Breast	Invasive ductal carcinoma	T1N1M0	3	IIA	Malignant	+	-	
A2	2	40	F	Breast	Invasive ductal carcinoma	T1N1M0	2	IIA	Malignant	+	-	[illegible]
A3	3	45	F	Breast	Invasive ductal carcinoma	T2N0M0	2	IIA	Malignant	-	++	
A4	4	46	F	Breast	Invasive ductal carcinoma	T2N0M0	2	IIA	Malignant	+++	+++	
A5	5	69	F	Breast	Invasive ductal carcinoma	T2N1M0	2	IIB	Malignant	*	-	
A6	6	45	F	Breast	Invasive ductal carcinoma	T1N0M0	2	IA	Malignant	+++	+++	[illegible]
A7	7	45	F	Breast	Invasive ductal carcinoma	T2N1M0	2	IIB	Malignant	++	++	[illegible]
A8	8	37	F	Breast	Invasive ductal carcinoma	T2N0M0	2	IIA	Malignant	+++	+++	
A9	9	43	F	Breast	Invasive ductal carcinoma	T1N1M0	2	IIA	Malignant	+++	+	
A10	10	44	F	Breast	Invasive ductal carcinoma	T1N0M0	2	IA	Malignant	+++	+++	
B1	11	45	F	Breast	Invasive ductal carcinoma	T4N2M0	2	IIIB	Malignant	+++	+++	
B2	12	45	F	Breast	Invasive ductal carcinoma	T1N0M0	2	IA	Malignant	-	-	
B3	13	63	F	Breast	Invasive ductal carcinoma	T2N0M0	2	IIA	Malignant	+++	+++	
B4	14	65	F	Breast	Invasive ductal carcinoma	T2N0M0	2	IIA	Malignant	+++	+	[illegible]
B5	15	55	F	Breast	Invasive ductal carcinoma	T3N2M0	2	IIIA	Malignant	+++	+++	
B6	16	46	F	Breast	Invasive ductal carcinoma	T1N0M0	3	IA	Malignant	*	*	
B7	17	44	F	Breast	Invasive ductal carcinoma	T2N0M0	2	IIA	Malignant	-	-	[illegible]
B8	18	34	F	Breast	Invasive ductal carcinoma	T4N0M0	2	IIIB	Malignant	+++	+	
B9	19	48	F	Breast	Invasive ductal carcinoma	T2N0M0	2	IIA	Malignant	+++	+++	[illegible]
B10	20	62	F	Breast	Invasive ductal carcinoma	T3N1M0	2	IIB	Malignant	+++	-	
C1	21	39	F	Breast	Invasive ductal carcinoma	T2N0M0	2	IIA	Malignant	+++	-	
C2	22	67	F	Breast	Invasive ductal carcinoma	T2N0M0	2	IIA	Malignant	+++	++	

C3	23	55	F	Breast	Invasive ductal carcinoma	T2N0M0	2	IIA	Malignant	+++	+	
C4	24	57	F	Breast	Invasive ductal carcinoma	T2N0M0	2	IIA	Malignant	+++	+++	[illegible]
C5	25	63	F	Breast	Invasive ductal carcinoma	T2N0M0	2	IIA	Malignant	+++	-	
C6	26	39	F	Breast	Invasive ductal carcinoma	T2N1M0	2	IIB	Malignant	+++	-	[illegible]
C7	27	42	F	Breast	Invasive ductal carcinoma	T2N0M0	2	IIA	Malignant	+++	+++	
C8	28	40	F	Breast	Invasive ductal carcinoma	T2N0M0	2	IIA	Malignant	+++	+++	[illegible]
C9	29	42	F	Breast	Invasive ductal carcinoma	T2N0M0	2	IIA	Malignant	+	-	[illegible]
C10	30	45	F	Breast	Invasive ductal carcinoma	T2N1M0	2	IIB	Malignant	+++	-	[illegible]
D1	31	52	F	Breast	Invasive ductal carcinoma	T3N0M0	2	IIB	Malignant	+++	+	
D2	32	42	F	Breast	Invasive ductal carcinoma	T2N1M0	3	IIB	Malignant	-	-	
D3	33	48	F	Breast	Invasive ductal carcinoma	T2N0M0	3	IIA	Malignant	+++	+++	
D4	34	54	F	Breast	Invasive ductal carcinoma	T2N1M0	2	IIB	Malignant	+++	+	
D5	35	46	F	Breast	Invasive ductal carcinoma	T3N0M0	3	IIB	Malignant	+++	+++	[illegible]
D6	36	68	F	Breast	Invasive ductal carcinoma	T2N0M0	2	IIA	Malignant	+++	+++	
D7	37	53	F	Breast	Invasive ductal carcinoma	T2N0M0	2	IIA	Malignant	-	-	
D8	38	58	F	Breast	Invasive ductal carcinoma	T2N1M0	3	IIB	Malignant	-	-	
D9	39	42	F	Breast	Invasive ductal carcinoma	T3N0M0	2	IIB	Malignant	-	-	[illegible]
D10	40	34	F	Breast	Intraductal carcinoma with early infiltration	T3N0M0	-	IIB	Malignant	+++	+	[illegible]
E1	41	47	F	Breast	Invasive ductal carcinoma	T3N0M0	2	IIB	Malignant	+++	+++	
E2	42	42	F	Breast	Invasive ductal carcinoma	T3N0M0	3	IIB	Malignant	-	-	[illegible]
E3	43	48	F	Breast	Invasive ductal carcinoma	T4N2M0	2	IIIB	Malignant	+++	+++	
E4	44	32	F	Breast	Invasive ductal carcinoma	T4N2M0	2	IIIB	Malignant	+++	+	
E5	45	52	F	Breast	Invasive ductal carcinoma	T2N1M0	2	IIB	Malignant	-	-	
E6	46	37	F	Breast	Invasive ductal carcinoma	T2N1M0	3	IIB	Malignant	-	-	
E7	47	74	F	Breast	Invasive ductal carcinoma	T2N0M0	3	IIA	Malignant	-	-	
E8	48	38	F	Breast	Invasive ductal carcinoma	T2N1M0	2	IIB	Malignant	-	-	[illegible]
E9	49	48	F	Breast	Invasive ductal carcinoma	T2N1M0	2	IIB	Malignant	+++	+++	
E10	50	51	F	Breast	Invasive ductal carcinoma	T3N1M0	3	IIIA	Malignant	-	-	[illegible]
F1	51	52	F	Breast	Invasive ductal carcinoma	T2N0M0	2	IIA	Malignant	+++	-	[illegible]
F2	52	51	F	Breast	Invasive ductal carcinoma	T2N2M0	2	IIIA	Malignant	-	-	[illegible]
F3	53	47	F	Breast	Invasive ductal carcinoma	T3N2M0	2	IIIA	Malignant	-	-	
F4	54	49	F	Breast	Invasive ductal carcinoma	T2N0M0	2	IIA	Malignant	+++	+++	
F5	55	46	F	Breast	Invasive ductal carcinoma	T4N0M0	2	IIIB	Malignant	+++	+++	
F6	56	39	F	Breast	Invasive ductal carcinoma	T4N0M0	2	IIIB	Malignant	-	-	[illegible]
F7	57	45	F	Breast	Invasive ductal carcinoma	T4N1M0	2	IIIB	Malignant	+++	-	
F8	58	50	F	Breast	Invasive ductal carcinoma	T4N1M0	2	IIIB	Malignant	+	-	
F9	59	49	F	Breast	Invasive ductal carcinoma	T2N0M0	2	IIA	Malignant	+++	+++	
F10	60	46	F	Breast	Invasive ductal carcinoma	T4N2M0	3	IIIB	Malignant	+++	-	
G1	61	60	F	Breast	Invasive ductal carcinoma	T1N0M0	2	IA	Malignant	++	+	
G2	62	49	F	Breast	Invasive ductal carcinoma	T2N0M0	2	IIA	Malignant	-	-	
G3	63	50	F	Breast	Invasive ductal carcinoma	T2N1M0	2	IIB	Malignant	+++	+	
G4	64	64	F	Breast	Invasive ductal carcinoma	T2N0M0	2	IIA	Malignant	-	-	[illegible]
G5	65	59	F	Breast	Invasive ductal carcinoma	T2N0M0	3	IIA	Malignant	+	-	
G6	66	38	F	Breast	Invasive ductal carcinoma	T3N1M0	3	IIIA	Malignant	-	-	
G7	67	54	F	Breast	Invasive ductal carcinoma	T2N2M0	3	IIIA	Malignant	+	-	[illegible]
G8	68	53	F	Breast	Invasive ductal carcinoma	T2N0M0	3	IIA	Malignant	+	-	
G9	69	40	F	Breast	Invasive ductal carcinoma	T2N0M0	2	IIA	Malignant	+	-	[illegible]
G10	70	46	F	Breast	Invasive ductal carcinoma	T2N0M0	2	IIA	Malignant	-	+	
H1	71	70	F	Breast	Invasive ductal carcinoma	T2N0M0	2	IIA	Malignant	-	-	
H2	72	27	F	Breast	Invasive ductal carcinoma	T2N1M0	3	IIB	Malignant	+++	-	[illegible]
H3	73	48	F	Breast	Invasive ductal carcinoma	T4N0M0	3	IIIB	Malignant	+++	+++	
H4	74	39	F	Breast	Invasive ductal carcinoma	T2N1M0	3	IIB	Malignant	+++	+++	
H5	75	52	F	Breast	Invasive ductal carcinoma	T4N2M0	3	IIIB	Malignant	-	-	[illegible]
H6	76	48	F	Breast	Invasive ductal carcinoma	T2N1M0	3	IIB	Malignant	+++	-	
H7	77	40	F	Breast	Invasive ductal carcinoma	T1N0M0	3	IA	Malignant	++	+	[illegible]
H8	78	47	F	Breast	Invasive ductal carcinoma	T2N0M0	3	IIA	Malignant	-	-	[illegible]
H9	79	41	F	Breast	Invasive ductal carcinoma	T2N0M0	3	IIA	Malignant	-	-	[illegible]
H10	80	53	F	Breast	Invasive ductal carcinoma	T3N0M0	3	IIB	Malignant	-	-	
I1	81	51	F	Breast	Invasive ductal carcinoma	T3N1M0	3	IIIA	Malignant	-	-	[illegible]
I2	82	32	F	Breast	Invasive ductal carcinoma	T2N0M0	3	IIA	Malignant	-	-	
I3	83	68	F	Breast	Invasive ductal carcinoma	T2N0M0	3	IIA	Malignant	+++	-	
I4	84	31	F	Breast	Invasive ductal carcinoma	T2N0M0	3	IIA	Malignant	-	-	[illegible]
I5	85	47	F	Breast	Invasive ductal carcinoma	T2N0M0	3	IIA	Malignant	+++	+	
I6	86	34	F	Breast	Invasive ductal carcinoma	T2N0M0	3	IIA	Malignant	-	-	
I7	87	56	F	Breast	Invasive ductal carcinoma	T2N0M0	3	IIA	Malignant	+++	+	
I8	88	37	F	Breast	Invasive ductal carcinoma	T2N1M0	3	IIB	Malignant	-	-	[illegible]
I9	89	43	F	Breast	Invasive ductal carcinoma	T2N1M0	2	IIB	Malignant	+++	+	
I10	90	45	F	Breast	Invasive ductal carcinoma	T2N0M0	3	IIA	Malignant	-	-	
J1	91	58	F	Breast	Invasive ductal carcinoma	T3N1M0	3	IIIA	Malignant	+	-	
J2	92	38	F	Breast	Invasive ductal carcinoma	T2N0M0	3	IIA	Malignant	-	-	
J3	93	53	F	Breast	Invasive ductal carcinoma	T2N0M0	3	IIA	Malignant	-	-	[illegible]
J4	94	32	F	Breast	Invasive ductal carcinoma	T1N1M0	2	IIA	Malignant	+++	+	[illegible]
J5	95	52	F	Breast	Invasive ductal carcinoma	T3N0M0	3	IIB	Malignant	-	-	[illegible]

J6	96	43	F	Breast	Invasive ductal carcinoma	T2N1M0	3	IIB	Malignant	-	-	[illegible]
J7	97	47	F	Breast	Invasive ductal carcinoma	T2N1M0	3	IIB	Malignant	++	++	
J8	98	30	F	Breast	Invasive ductal carcinoma	T2N2M0	3	IIIA	Malignant	-	-	[illegible]
J9	99	46	F	Breast	Invasive ductal carcinoma	T4N0M0	3	IIIB	Malignant	-	-	
J10	100	45	F	Breast	Invasive ductal carcinoma	T3N1M0	3	IIIA	Malignant	++	+	[illegible]
K1	101	43	F	Breast	Adjacent normal breast tissue	-	-	-	NAT	+	+	
K2	102	56	F	Breast	Cancer adjacent breast tissue (adenosis with epithelial hyperplasia)	-	-	-	AT	+	+	
K3	103	40	F	Breast	Adjacent normal breast tissue	-	-	-	NAT	+	+	
K4	104	41	F	Breast	Adjacent normal breast tissue	-	*	-	NAT	+	+	
K5	105	38	F	Breast	Adjacent normal breast tissue	-	-	-	NAT	+	+	
K6	106	29	F	Breast	Adjacent normal breast tissue	-	-	-	NAT	*	*	
K7	107	41	F	Breast	Adjacent normal breast tissue	-	*	-	NAT	+++	++	
K8	108	42	F	Breast	Adjacent normal breast tissue	-	-	-	NAT	+++	+++	
K9	109	35	F	Breast	Adjacent normal breast tissue	-	-	-	NAT	+++	+	
K10	110	39	F	Breast	Adjacent normal breast tissue	-	-	-	NAT	*	*	
-	0	42	M	Adrenal gland	Pheochromocytoma (tissue marker)		-		Malignant			

TNM grading:

T - Primary tumor
Tx - Primary tumor cannot be assessed
T0 - No evidence of primary tumor
Tis - Carcinoma in situ; intraepithelial or invasion of lamina propria
T1 - Tumor invades submucosa
T2 - Tumor invades muscularis propria
T3 - Tumor invades through muscularis propria into subserosa or into non-peritonealized pericolic or perirectal tissues.
T4 - Tumor directly invades other organs or structures and/or perforate visceral peritoneum

N - Regional lymph nodes
Nx - Regional lymph nodes cannot be assessed
N0 - No regional lymph node metastasis
N1 - Metastasis in 1 to 3 regional lymph nodes
N2 - Metastasis in 4 or more regional lymph nodes

M - Distant metastasis
Mx - Distant metastasis cannot be assessed
M0 - No distant metastasis
M1 - Distant metastasis
